# PINLYP-mediated phospholipid metabolism reprogramming contributes to chronic herpesvirus infection

**DOI:** 10.1371/journal.ppat.1013146

**Published:** 2025-05-15

**Authors:** Zhangmengxue Lei, Wendi Wei, Mingyu Wang, Yun Xu, Lei Bai, Ying Gao, Congwei Jiang, Fangxia Li, Na Tian, Linlin Kuang, Ruiliang Zhu, Gang Pang, Ke Lan, Suihan Feng, Xiaozhen Liang

**Affiliations:** 1 University of Chinese Academy of Sciences, Shanghai Institute of Immunity and Infection, Chinese Academy of Sciences, Shanghai, China; 2 State Key Laboratory of Virology, Modern Virology Research Center, College of Life Sciences, Wuhan University, Wuhan, China; The University of North Carolina at Chapel Hill School of Medicine, UNITED STATES OF AMERICA

## Abstract

Many viruses alter the phospholipid metabolism to benefit their own life cycles. It is unclear whether the host or the virus is driving phospholipid metabolism reprogramming, and how virus infections are affected by the metabolic status. Here we report that phospholipase A2 inhibitor and LY6/PLAUR domain-containing protein (PINLYP) inhibits Kaposi’s sarcoma-associated herpesvirus (KSHV) lytic reactivation by remodeling phospholipid metabolism and especially triacylglycerol (TAG) biosynthesis. PINLYP deficiency led to increased phospholipase cPLA2α activity, cPLA2α-mediated AKT phosphorylation, and KSHV lytic reactivation. Analyses of RNA-seq and lipidomics reveal that PINLYP regulates long-chain fatty acid CoA ligase ACSL5 expression and TAG production. The inhibition of ACSL5 activity or TAG biosynthesis suppresses AKT phosphorylation and KSHV lytic reactivation, restoring the phenotype of PINLYP deficiency. This finding underscores the pivotal role of PINLYP in remodeling phospholipid metabolism and promoting viral latency, which sheds new light on how phospholipid metabolism is regulated by herpesvirus and provides a potential target for controlling chronic herpesvirus infection.

## Author summary

Many viruses change the host phospholipid metabolism for their own life cycles. However, it remains unknown for the underlying mechanism by which phospholipid metabolism reprogramming is driven by the host or the virus. Here we report that KSHV infection induces the expression of phospholipase A2 inhibitor and LY6/PLAUR domain-containing protein PINLYP, which in turn remodels phospholipid metabolism and particularly TAG biosynthesis in KSHV latently infected cells, ultimately leading to the suppression of KSHV lytic reactivation. Our results reveal that KSHV hijacks the host factor PINLYP to change phospholipid metabolism for benefiting its latent infection, providing a new target for controlling herpesvirus infection.

## Introduction

Kaposi’s sarcoma-associated herpesvirus (KSHV) also known as human herpesvirus 8 (HHV-8) is associated with the development of neoplastic diseases such as Kaposi’s sarcoma (KS), primary effusion lymphoma (PEL), and multicentric Castleman’s disease (MCD) [1,2]. Like other herpesviruses, KSHV typically undergoes a latent and lytic biphase life cycle [3], and both the latent and lytic phases are crucial for KSHV-associated pathogenesis and tumorigenesis. The replication and transcription activator (RTA) is a key KSHV lytic switch protein that triggers KSHV lytic reactivation, initiating the cascade of KSHV lytic gene expression, viral genome replication, and progeny virion production [4,5].

KSHV latency and reactivation are well regulated by both viral and cellular proteins as well as host-regulating pathways [6]. The PI3K-AKT signaling pathway is shown to be closely associated with the life cycle of KSHV and the etiology of associated cancers [7–9]. KSHV *de novo* infection activates this PI3K-AKT signaling pathway, and several viral proteins have also been reported to regulate the PI3K-AKT signaling pathway [10,11]. K1 promotes the phosphorylation of the PI3K regulatory subunit p85 and inactivates the negative regulator PTEN to activate the AKT signal [12–14]. KSHV vGPCR directly activates AKT in a PI3K-dependent manner [7,15,16]. KSHV vIL-6 activates the AKT pathway by activating gp130 and downregulating caveolin 1. Activation of the PI3K-AKT signaling pathway is indispensable for the KSHV lytic replication process [13,17], contributing to KSHV latency [18].

The phosphorylation and activation of AKT are tightly associated with the lipid molecule ceramide, which is related to phospholipid metabolism and triglyceride (TAG) biosynthesis [19]. Cytosolic phospholipase A2 α (cPLA2α) is one of the most important intracellular lipolytic enzymes that hydrolyze the phospholipids to free fatty acids (FAs) and lysophospholipids [20,21], which provide lipid mediator production and substrates for *de novo* TAG synthesis in endoplasmic reticulum by lipogenesis. Long-chain acyl-CoA synthetase (ACSL) mediates the thioesterification of FAs to produce long-chain acyl-CoAs (Acyl-CoA), initiating the formation of diacylglycerol (DAG) and subsequent biosynthesis of TAG [22]. Dynamic lipidome alterations are well documented during viral infection [23–25]. Viruses can reprogram the phospholipid metabolism of the host cells in favor of their replication and life cycles [26–29]. KSHV latent infection also induces host cell lipogenesis, and fatty acid synthesis is required for the survival of KSHV-latently infected endothelial cells [30].

Phospholipase A2 inhibitor and LY6/PLAUR domain-containing protein (PINLYP) belongs to the lymphocyte antigen-6 (Ly6)/urokinase-type plasminogen activator receptor (uPAR) superfamily, whose functions are diverse, including cell proliferation, migration, immune cell maturation, macrophage activation, and cytokine generation [31–34]. Our previous work reported that PINLYP interacts with TBK1, induces type I interferon (IFN) production, and protects the host against viral infection [35]. Here, we uncovered the crucial role of PINLYP in phospholipid metabolism reprogramming, especially in TAG biosynthesis during KSHV latent infection. It alters the phospholipid landscape and primarily TAG biosynthesis by reprogramming phospholipid metabolism instead of influencing *de novo* TAG biosynthesis, which ultimately mediates AKT activation and KSHV latency by inhibiting viral lytic reactivation.

## Results

### PINLYP deficiency promotes KSHV lytic reactivation

**Fig 1:**
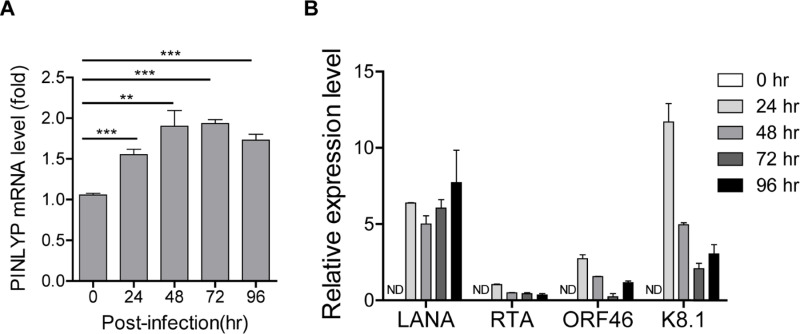
KSHV *de novo* infection induces PINLYP expression. HUVEC cells were infected with KSHV, total RNA was isolated from the infected cells at the indicated time and subjected to qRT-PCR analyses. (A) Detection of PINLYP mRNA by normalizing to GAPDH. Error bars indicated SEM, *p < 0.05, **p < 0.01, ***p < 0.001, two-tailed unpaired *t*-test. (B) Detection of KSHV LANA, RTA, ORF46, and K8.1 mRNA expression by normalizing to GAPDH. ND represents not detectable.

We previously characterized a new gene PINLYP that was identified in a murine gammaherpesvirus 68 (MHV68)-associated lymphoma model and showed that PINLYP positively regulates type I IFN innate immunity and protects the host against virus infection [35]. To further investigate the function of PINLYP during gammaherpesvirus latent infection, we measured the kinetics of PINLYP expression during *de novo* infection of HUVEC with KSHV since KSHV infection of HUVEC undergoes a temporary lytic phase at the early stage of infection and ultimately establishes tight latency at the late stage [36]. Notably, KSHV *de novo* infection of HUVEC resulted in a gradual increase of PINLYP expression by 72 h post-infection (Fig 1A), accompanied by a gradual decrease of KSHV RTA, ORF46, and K8.1 lytic gene expression (Fig 1B). This indicates that KSHV induces PINLYP expression during the temporary lytic phase of *de novo* infection and that PINLYP induction reaches a steady state when KSHV starts the entry into the latent phase.

**Fig 2:**
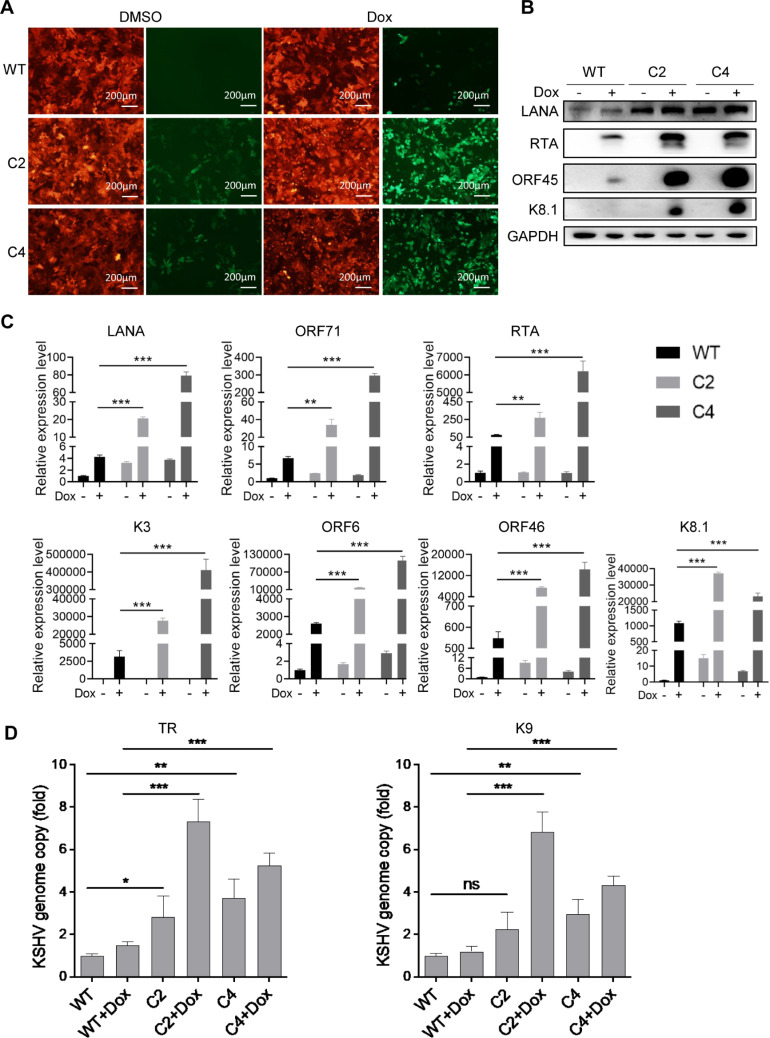
PINLYP deficiency promotes KSHV lytic reactivation from latency. Wild-type (WT) and PINLYP KO (clones C2 and C4) iSLK-RGB cells were treated with DMSO or Dox (1 μg/mL) for 24 h. (A) Fluorescence imaging showed RFP-positive KSHV infected cells and GFP-positive KSHV-reactivating cells. Each image was collected from three to five represented fields of three biological replicates for every sample. Image magnification: 10 ×. (B) Whole cell lysate was prepared from the treated cells and western blot analyses were performed with the indicated antibodies. (C) qRT-PCR analyses were performed with the specific primers corresponding to KSHV LANA, ORF71, RTA, K3, ORF6, ORF46, and K8.1. Viral gene mRNA was normalized to GAPDH mRNA. All error bars indicated SEM, *p < 0.05, **p < 0.01, ***p < 0.001, two-tailed unpaired *t*-test. (D) Genomic DNA was isolated from treated cells, and quantitative PCR analyses were performed to detect KSHV genome copy using the primers corresponding to TR and K9. Viral genomic DNA was normalized to GAPDH genomic DNA. All error bars indicated SEM, *p < 0.05, **p < 0.01, ***p < 0.001, ns represents not significant. Two-tailed unpaired *t*-test.

To address the role of PINLYP during KSHV latent infection, we used the CRISPR/Cas9 gene editing system to generate PINLYP knockout (KO) iSLK.RGB cells, which harbor latent KSHV reporter virus that contains a doxycycline (Dox)-inducible RTA to enable entry into the lytic cycle and marks red fluorescent latent, green fluorescent immediate early, and blue fluorescent late viral gene expression. Two distinct clones, C2 and C4, of PINLYP KO cells derived from diverse sgRNAs, were selected and sequenced, and the knockout efficiency was further confirmed by real-time quantitative PCR and immunoblot analyses (S1A Fig). A modest number of GFP-positive KSHV reactivating cells was observed in PINLYP KO cells even prior to Dox treatment (Fig 2A), indicating that PINLYP expression is crucial for KSHV maintenance of tight latency. After Dox-induced KSHV lytic reactivation, PINLYP deficiency increased the number of GFP-positive cells (Fig 2A), elevated the level of KSHV proteins RTA, ORF45, and K8.1 (Fig 2B), and upregulated the mRNA level of KSHV viral genes LANA, ORF71, RTA, K3, ORF6, ORF46, and K8.1 (Fig 2C). Consistently, the number of KSHV viral genome copies was significantly higher in PINLYP KO cells compared to WT cells during KSHV lytic reactivation (Fig 2D). The amount of KSHV progeny viruses released from the Dox-treated cells was further examined by collecting the supernatant to re-infect 293T cells. In line with the above observation, there were more RFP-positive, KSHV-infected 293T cells after infection with the supernatant from Dox-treated PINLYP KO cells as compared to the cells infected with the supernatant from WT cells (S1B and S1C Fig), suggesting PINLYP deficiency leads to increased viral production during KSHV lytic reactivation. Altogether, these results demonstrate that PINLYP inhibits KSHV lytic reactivation from latency.

### Overexpression of PINLYP suppresses KSHV lytic reactivation from latency

**Fig 3:**
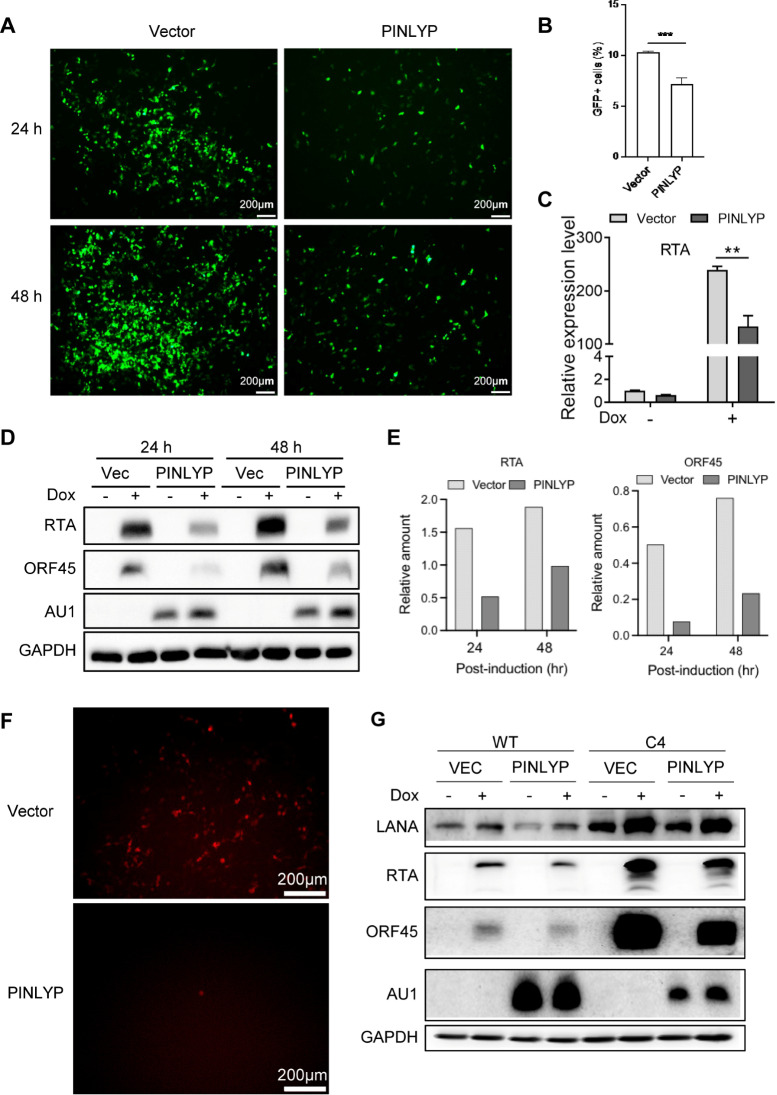
Overexpression of PINLYP inhibits KSHV lytic reactivation from latency. (A) iSLK-RGB cells were transfected with vector (Vec) alone or PINLYP-AU1-expressing plasmid for 24 h and subsequently treated with Dox (1 μg/mL) for 24 and 48 h. Fluorescence imaging showed GFP-positive KSHV reactivating iSLK-RGB cells. Image magnification: 5 × . (B) Quantitative analyses of GFP-positive KSHV reactivating iSLK-RGB cells at 48 h post-transfection by FACS analyses for (A). Each sample had three replicates (two experiments). Unpaired *t*-test data. Error bars indicated SEM, *p < 0.05, **p < 0.01, ***p < 0.001. (C) Same as A, total RNA was prepared after Dox treatment for 24 h and subsequently subjected to qRT-PCR analyses using RTA specific primers. RTA mRNA was normalized to GAPDH mRNA. Unpaired *t*-test da*t*a. Error bars indicated SEM, *p < 0.05, **p < 0.01, ***p < 0.001. (D) Same as A, whole cell lysates were prepared and subsequently subjected to immunoblot analyses with the indicated antibodies. (E) Quantitative analyses of RTA and ORF45 protein levels for (D). (F) Same as A, Supernatant were collected from Dox-treated iSLK-RGB cells for 48 h and used to infect 293T cells, fluorescence imaging showed RFP-positive KSHV-infected 293T cells at 72 h post-infection. Image magnification: 10 × . (G) WT and PINLYP KO (C4) iSLK-RGB cells were transfected with vector alone or PINLYP-AU1-expressing plasmid for 48 h and subsequently treated with DMSO or Dox (1 μg/mL) for 24 h, followed by immunoblot analyses with the indicated antibodies.

To further verify PINLYP inhibition of KSHV lytic reactivation, iSLK.RGB cells were transfected with a vector alone or a PINLYP-AU1-expressing plasmid. Overexpression of PINLYP significantly reduced the number of GFP-positive KSHV reactivating cells (Fig 3A and 3B), decreased the mRNA expression of KSHV lytic gene RTA (Fig 3C), and downregulated the protein expression of KSHV lytic genes RTA and ORF45 at 24 and 48 h post-Dox induction (hpi) (Fig 3D and 3E). To analyze the progeny virus production, we collected the supernatant from transfected cells at 48 hpi to infect 293T cells. When compared with the 293T cells infected with the supernatant from vector-transfected iSLK.RGB cells, a significant lower number of RFP-positive 293T cells were observed after infection with the supernatant from PINLYP-transfected iSLK.RGB cells (Fig 3F), suggesting that PINLYP overexpression results in a decrease in infectious virion production in Dox-treated iSLK.RGB cells. In addition, the ectopic expression of PINLYP markedly decreased the protein expression of KSHV lytic genes RTA and ORF45 in Dox-treated PINLYP KO cells, restoring the phenotype resulting from PINLYP deficiency (Fig 3G). These results confirm that PINLYP could efficiently inhibit KSHV lytic reactivation from latency.

### PINLYP inhibition of KSHV lytic reactivation is via PI3K-AKT signaling pathway

RTA is the master transcription activator that triggers the KSHV lytic cycle from latency. We next determined whether PINLYP could directly regulate the activation of RTA. 293T cells were transfected with RTA luciferase promoter plasmid in the presence or absence of PINLYP-expressing plasmid. The luciferase assay showed that no significant difference was observed for the RTA luciferase activity between PINLYP-expressing cells and control cells (S2 Fig). KSHV latent protein LANA plays a crucial role in the establishment and maintenance of viral latency; it can interfere with the transactivation capabilities of RTA and suppress RTA-triggered lytic reactivation [5]. Next, we examined whether PINLYP would regulate LANA suppression of RTA activity. 293T cells were co-transfected with LANA-Flag and PINLYP-AU1 plasmids, together with the RTA luciferase promoter reporter. Consistent with the previous report, LANA expression alone could inhibit the activity of RTA promoter; however, PINLYP co-expression failed to show any apparent effect on LANA inhibition of RTA activation (S2 Fig), suggesting that PINLYP neither directly intervenes in the activation of RTA nor interferes with LANA suppression of RTA activity. This data rules out the likelihood that PINLYP inhibits KSHV lytic reactivation by regulating RTA activity or LANA suppression of RTA activity.

**Fig 4:**
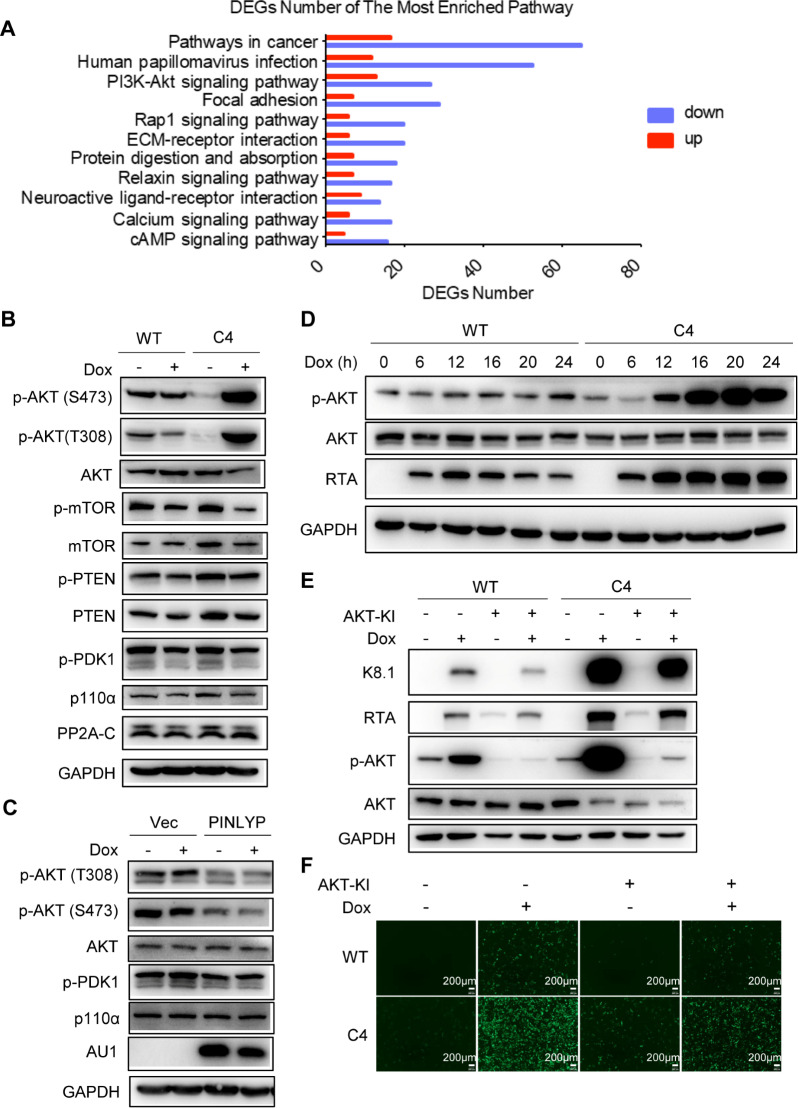
PINLYP deficiency promotes KSHV lytic reactivation via PI3K-AKT signaling pathway. (A) Gene Ontology analysis of differentially expressed genes (DEGs) in WT and PINLYP KO (C4) iSLK-RGB cells. (B) WT and PINLYP KO (C4) iSLK-RGB cells were treated with Dox (1 μg/mL) for 24 h and subjected to immunoblot analyses with the indicated antibodies. (C) iSLK-RGB cells were transfected with vector (Vec) alone or PINLYP-AU1-expressing plasmid for 48 h and subsequently treated with Dox (1 μg/mL) for 24 h, followed by immunoblot analyses with the indicated antibodies. (D) WT and PINLYP KO (C4) iSLK-RGB cells were treated with Dox (1 μg/mL) for the indicated time, followed by immunoblot analyses with the indicated antibodies.(E) WT and PINLYP KO (C4) iSLK-RGB cells were treated with Dox (1 μg/mL) together with AKT specific inhibitor (AKT-KI, 10 μM) for 24 h, followed by immunoblot analyses with the indicated antibodies. (F) Same as E, representative fluorescence imaging showed iSLK-RGB cells with GFP-positive KSHV reactivating cells. Each image was collected from three to five represented fields of three biological replicates for every sample. Image magnification: 10 × .

To further explore the mechanism underlying PINLYP inhibition of KSHV lytic reactivation, we performed RNA-seq analyses for WT and PINLYP KO iSLK.RGB cells. A direct comparison of gene expression between WT and PINLYP KO cells revealed 987 significantly differentially expressed genes, including 236 upregulated and 751 downregulated genes (S3A Fig). A prominent enrichment of gene sets and pathways was related to the PI3K-AKT signaling pathway (Fig 4A), which has been shown to be vital for KSHV lytic reactivation. Immunoblot analyses were performed to detect the PI3K-AKT signaling pathway. Strikingly, PINLYP deficiency markedly increased the phosphorylation level of AKT (S473) by over fourfold and AKT (T308) by approximately sevenfold upon Dox-induced KSHV lytic reactivation (Figs 4B and S3B), whereas PINLYP overexpression decreased the phosphorylation level of AKT (S473) and AKT (T308) by about twofold in iSLK.RGB cells (Fig 4C), indicating that PINLYP inhibits AKT activation during KSHV lytic reactivation, which supports RNA-seq analyses. Time course analyses further revealed that the level of p-AKT began to markedly increase between 6 and 12 hpi, continued to increase by 16 hpi, and stayed at a high level by 24 hpi in PINLYP KO iSLK.RGB cells, which was concurrently accompanied by a similar change in RTA protein level (Figs 4D and S3C). This suggests that PINLYP deficiency upregulates AKT activation, which might contribute to enhanced KSHV lytic reactivation. In contrast, a trivial change was observed for p-AKT in WT cells after Dox treatment; RTA expression was induced at 6 hpi and reached its highest level at 12 hpi, followed by a gradual decrease at the later time point in WT cells (Fig 4D).

To further validate whether p-AKT elevation involves enhanced KSHV lytic reactivation in PINLYP KO cells, we used AKT-specific inhibitor (AKT-KI) to treat WT and PINLYP KO iSLK.RGB cells after measuring the cytotoxicity of AKT-KI (S3D Fig). A non-cytotoxic concentration of AKT-KI treatment led to a reduction of KSHV lytic proteins K8.1 and RTA in both WT and PINLYP KO cells after Dox-induced KSHV lytic reactivation (Fig 4E) and also resulted in a decrease of GFP-positive, KSHV-reactivating cell numbers in both WT and PINLYP KO cells (Fig 4F), confirming that AKT activation is not only important for KSHV lytic reactivation in WT cells but also critical for enhanced KSHV lytic reactivation driven by PINLYP deficiency. We recently showed that PINLYP mediates type I IFN anti-viral innate immunity [35]. Since IFN and IFN-induced proteins have been implicated in the control of KSHV latency [5], we further examined the IFN signaling pathway and showed that the p-IRF3 level was marginally upregulated in PINLYP KO cells, whereas the p-STAT1 level exhibited no distinction between WT and PINLYP KO cells during KSHV lytic reactivation (S3E Fig). Our RNA-seq analyses also revealed that IFN signaling was not among the gene sets and pathways enriched when compared between WT and PINLYP KO cells. It’s highly likely that PINLYP-mediated type I IFN signaling is not involved in PINLYP regulation of KSHV lytic reactivation. Collectively, these data suggest that PINLYP deficiency enhances KSHV lytic reactivation through activation of the PI3K-AKT signaling pathway.

### cPLA2α activation is required for PINLYP regulation of AKT phosphorylation and KSHV lytic reactivation

**Fig 5:**
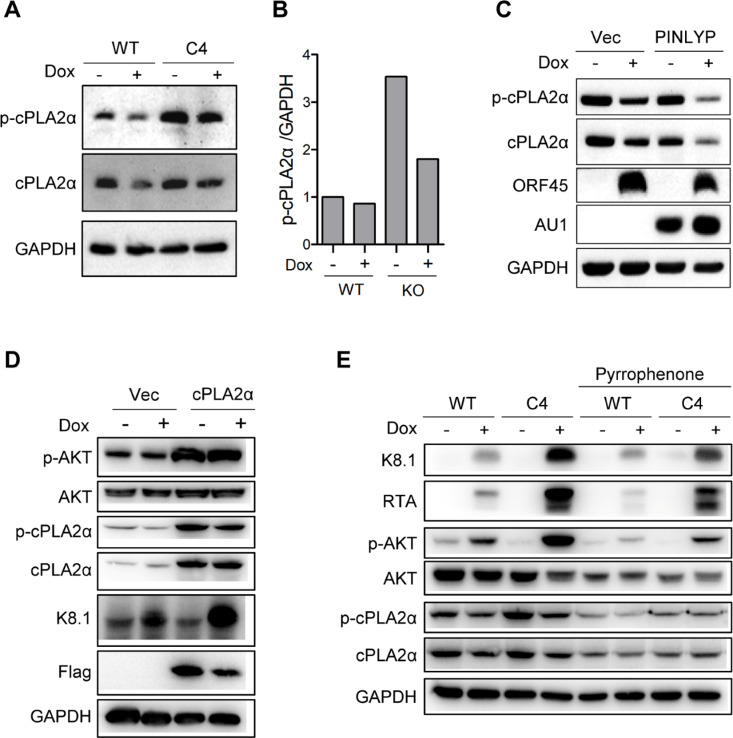
cPLA2α activation is required for increased AKT phosphorylation and KSHV lytic reactivation in PINLYP KO cells. (A) WT and PINLYP KO (C4) iSLK-RGB cells were treated with Dox (1 μg/mL) for 24 h and subjected to immunoblot analyses with specific antibodies as indicated. (B) Quantitation of p-cPLA2α for (A). (C) iSLK-RGB cells were transfected with vector alone (Vec) or PINLYP-AU1-expressing plasmid for 48 h and subsequently treated with Dox (1 μg/mL) for 24 h, followed by immunoblot analyses with specific antibodies as indicated. (D) iSLK-RGB cells were transfected with vector alone (Vec) or cPLA2α-expressing plasmid for 48 h and subsequently treated with Dox (1 μg/mL) for 24 h, followed by immunoblot analyses with specific antibodies as indicated. (E) WT and PINLYP KO (C4) iSLK-RGB cells were treated with Dox (1 μg/mL) in the presence or absence of cPLA2α inhibitor Pyrrophenone (8 μM) for 24 h and subjected to immunoblot analyses with specific antibodies as indicated.

It is worth noting that during KSHV lytic reactivation, no obvious difference was observed between WT and PINLYP KO cells for p-mTOR, p-PTEN, p-PDK1, and p110α that take part in the PI3K-AKT pathway and AKT phosphorylation (Fig 4B). Considering that PINLYP contains a PLA2 inhibitor domain and cPLA2α can activate the PI3K-AKT signaling pathway [37], we next tested whether an elevated activation of AKT driven by PINLYP deficiency is dependent on cPLA2α activity. The immunoblot analyses revealed that PINLYP deficiency increased the phosphorylation of cPLA2α during KSHV lytic reactivation (Fig 5A and 5B), while overexpression of PINLYP decreased the expression and phosphorylation of cPLA2α upon KSHV lytic reactivation in iSLK.RGB cells (Fig 5C), suggesting that PINLYP inhibits the activity of cPLA2α during KSHV lytic reactivation. Because of the coincidental inhibition of both cPLA2α and AKT by PINLYP, we reasoned that AKT activation might be regulated by cPLA2α activity in PINLYP KO iSLK.RGB cells during KSHV lytic reactivation. To test this, we transfected a cPLA2α-expressing plasmid into iSLK.RGB cells. Overexpression of cPLA2α obviously increased the level of p-AKT before and after Dox treatment and upregulated the expression of KSHV lytic gene K8.1 upon Dox-induced KSHV lytic reactivation (Fig 5D), indicating that cPLA2α could activate AKT in iSLK.RGB cells and simultaneously enhance viral lytic gene expression during KSHV reactivation. To further validate the role of cPLA2α in AKT activation and KSHV lytic reactivation, we used a cPLA2α-specific inhibitor, pyrrophenone, to treat WT and PINLYP KO iSLK.RGB cells. Strikingly, a non-toxic concentration of pyrrophenone treatment significantly reduced the level of p-AKT and of KSHV lytic proteins K8.1 and RTA in PINLYP KO cells upon Dox-induced KSHV lytic reactivation, almost completely restoring the phenotype in PINLYP KO cells similar to that observed in WT cells (Fig 5E). These data demonstrate that PINLYP deficiency promotes KSHV lytic reactivation via cPLA2α-mediated AKT phosphorylation.

### PINLYP deficiency increases ACSL5 expression and alters lipid landscape

**Fig 6:**
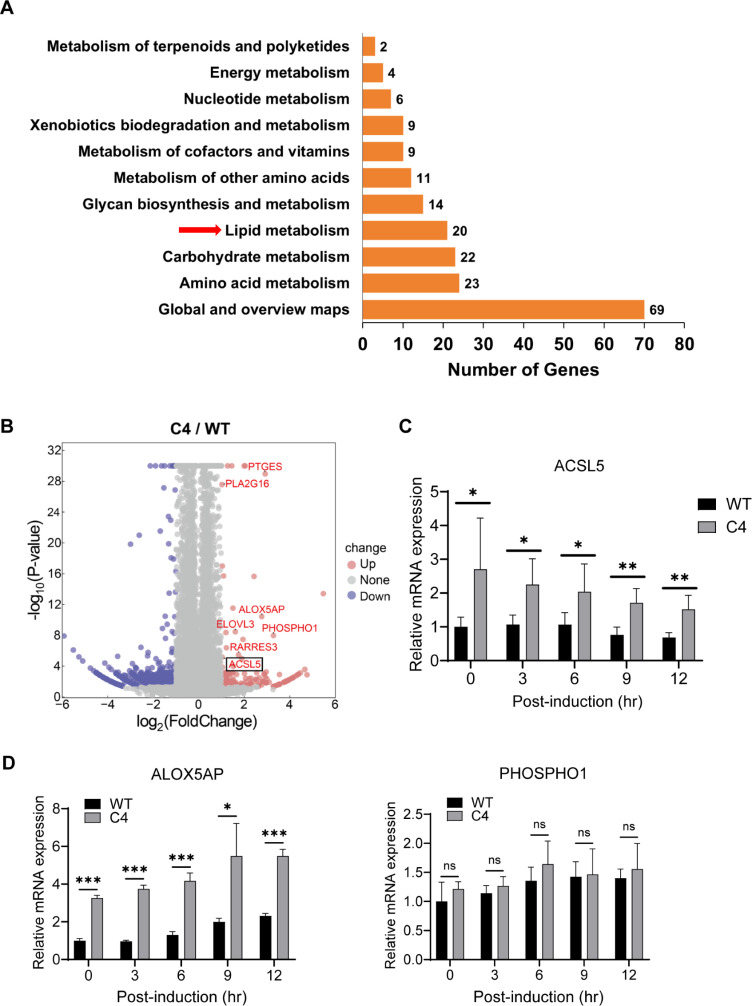
PINLYP deficiency increases ACSL5 expression. (A) Functional annotation and enrichment analyses of differentially expressed genes related to metabolism pathways for RNA-seq data from WT and PINLYP KO iSLK-RGB cells. (B) Volcano plot showed the differentially expressed genes related to phospholipid metabolism for RNA-seq data of WT and PINLYP KO iSLK-RGB cells. (C) WT and PINLYP KO (C4) iSLK-RGB cells were treated with Dox for the indicated time and subjected to qRT-PCR analyses with ACSL5-specific primers. ASCL5 mRNA was normalized to GAPDH mRNA. Unpaired *t*-test data are graphed on the y axis as fold change relative to WT cells. All error bars indicated SEM, *p < 0.05, **p < 0.01. (D) Same as (C) except that qRT-PCR analyses were performed with the specific primers corresponding to ALOX5AP and PHOSPHO1 genes. ALOX5AP and PHOSPHO1 mRNA were normalized to GAPDH mRNA. Unpaired *t*-test data. Error bars indicated SEM, *p < 0.05, **p < 0.01, ***p < 0.001, ns represents not significant.

Given that cPLA2α plays a crucial role in regulating phospholipid metabolism and is directly involved in phospholipid hydrolysis, we wonder whether cPLA2α-mediated phospholipid metabolism is involved in PINLYP regulation of AKT phosphorylation and KSHV lytic reactivation. To address this, we analyzed the RNA-seq data set and gene expression profile of WT and PINLYP KO iSLK.RGB cells. The gene ontology and pathway enrichment revealed that lipid metabolism, carbohydrate metabolism, and amino acid metabolism were among the top three metabolism processes with the highest number of differentially expressed genes between WT and PINLYP KO cells (Fig 6A). There were 20 differentially expressed genes related to lipid metabolism, long-chain fatty acid CoA ligase 5 (ACSL5), 5-lipoxygenase-activating protein (ALOX5AP), and Phosphoethanolamine/phosphocholine phosphatase (PHOSPHO1) were among the upregulated genes in PINLYP KO cells (Fig 6B). Real-time quantitative PCR analyses confirmed that mRNA expression of ACSL5 and ALOX5AP was significantly higher in PINLYP KO cells as compared to WT cells regardless of Dox treatment, but the expression of PHOSPHO1 that is an enzyme implicated in bone and cartilage formation showed a similar level between WT and PINLYP KO cells (Fig 6C and 6D).

ACSL5 is a member of the long-chain fatty acid coenzyme A ligase family that converts free long-chain fatty acids into Acyl-CoA, which is utilized for phospholipid biosynthesis. Functionally, ACSL5 is important in channeling fatty acids for the production of TAG [38] and 1-O-acylceramide synthesis [39]. The heightened expression of ACSL5 in PINLYP KO cells suggested that PINLYP might play a role in regulating lipid metabolism and TAG biosynthesis.

**Fig 7:**
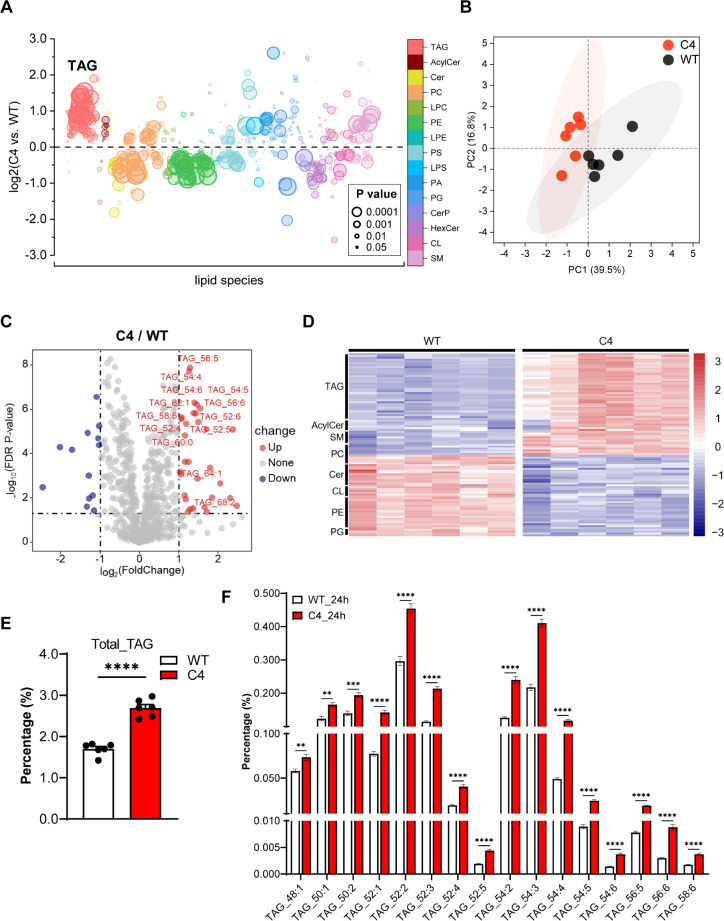
PINLYP deficiency alters phospholipid landscape and increases TAG biosynthesis. (A) Bubble plot showed the comparison of lipid species abundance between WT and PINLYP KO cells. The circle size is proportionate to *p* value. Each data point represents a lipid species. (B) The score plot of principal component analysis (PCA) of lipidomic profiles separated WT (black) and PINLYP KO cells (red). (C) Volcano plot of the lipid molecular species altered in PINLYP KO vs WT iSLK-RGB cells. The log2 fold change of PINLYP KO vs. WT group was plotted against the -log10 *p*-value. Statistical significance was evaluated by *t*-test (*p*-value < 0.05) and dots in red and blue represent significantly altered lipid species, respectively. (D) Heat map of lipid subclasses with altered level in PINLYP KO (C4) vs WT cells. Rows represent lipid metabolites and columns are sample replicates. (E) Plots showed statistical analyses of total TAG. All error bars indicated SEM, *p < 0.05, **p < 0.01, ***p < 0.001, ****p < 0.0001, two-tailed unpaired *t*-test. (F) Quantitative lipidomic analyses of TAG subclasses in WT and PINLYP KO iSLK-RGB cells (C4) (six replicates for each sample). All error bars indicated SEM, *p < 0.05, **p < 0.01, ***p < 0.001, ****p < 0.0001, two-tailed unpaired *t*-test.

To confirm the role of PINLYP in phospholipid metabolism and TAG biosynthesis, we performed lipidomic analyses for WT and PINLYP KO iSLK.RGB cells. A total of 15 lipid subclasses and 698 lipid species were detected and quantified. Phosphatidyl choline (PC), TAG, phosphatidylethanolamine (PE), and phosphatidyl serine (PS) were among the top lipid species with the highest number of detected lipid subclasses (Figs 7E and S4). Further examination of the lipidomic data using a bubble plot and a principal component analysis showed a clear difference between PINLYP KO and WT cells (Fig 7A and 7B). This indicated that PINLYP deficiency induced changes in the phospholipid profile. It is remarkable that TAG exhibited the greatest relative upregulation in PINLYP KO cells in comparison to WT cells (Fig 7C-7F), implicating that PINLYP deficiency might predominantly enhance the diacylglycerol acyltransferase (DGAT)-catalyzed TAG biosynthesis pathway. Meanwhile, a slight increase of 1-O-acylceramides (AcylCer) and a decrease of ceramide species were also observed (S4 Fig), in line with a previous report that ACSL5 supports AcylCer production [39], though the effect is rather moderate compared to TAG biosynthesis. The other upregulated lipid was PC, whereas PE, cardiolipin (CL), and phosphatidyl glycerol (PG) were downregulated in PINLYP KO cells when compared with WT cells (S4 Fig). Altogether, these results showed that cells lacking PINLYP undergo lipid remodeling by reshuffling lipid metabolism related genes such as ACSL5, leading to a significant increase in TAG and demonstrating the essential role of PINLYP in maintaining lipid homeostasis.

### PINLYP inhibition of KSHV lytic reactivation is dependent on PINLYP-mediated phospholipid metabolism and TAG biosynthesis

**Fig 8:**
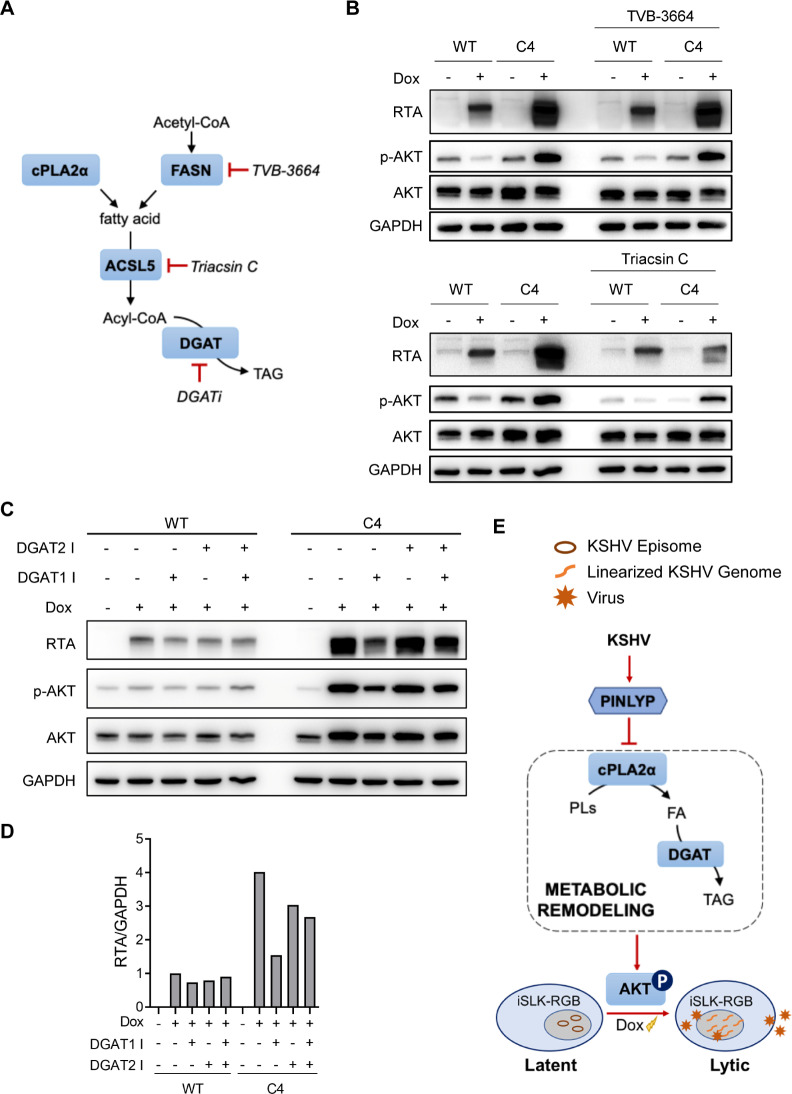
Increased biosynthesis of TAG is responsible for enhanced AKT activation and KSHV lytic reactivation in PINLYP KO cells. (A) Schematic pathway of TAG biosynthesis. (B) WT and PINLYP KO (C4) iSLK-RGB cells were treated with Dox in the presence of FASN inhibitor TVB-3664 (5 μM) or ACSL5 inhibitor Triacsin C (3 μM) for 24 h, followed by immunoblot analyses with specific antibodies as indicated. (C) WT and PINLYP KO (C4) iSLK-RGB cells were treated with Dox (1 μg/mL) in the presence or absence of DGAT inhibitor DGAT1 I (50 μM) or DGAT2 I (50 μM) for 24 h, followed by immunoblot analyses with the specific antibodies as indicated. (D) Quantitative analysis of RTA expression for (C). (E) Model for the effect of PINLYP-mediated phospholipid metabolism on KSHV latent infection. KSHV induces PINLYP expression during the establishment of viral latency. PINLYP in turn remodels phospholipid metabolism by inhibiting cPLA2α activity and subsequently regulates AKT phosphorylation, which promotes the inhibition of KSHV lytic reactivation and maintenance of KSHV latency.

PINLYP deficiency resulted in systemic lipid metabolic remodeling in PINLYP KO iSLK.RGB cells, but it is unclear whether this metabolic reshuffle is functionally important to KSHV lytic reactivation. Given the significant variances in total TAG abundance between WT and PINLYP KO cells, we investigated whether blocking the TAG biosynthetic pathway has any effect on KSHV lytic reactivation. As a major building block for TAG synthesis, fatty acid can be produced after lipid remodeling or synthesized from acetyl-CoA through fatty acid synthases (FASN). We used a selective fatty acid synthase inhibitor TVB-3664 and a potent Acyl-CoA synthetase inhibitor triacsin C to treat WT and PINLYP KO cells after measuring the cytotoxicity of the inhibitors (S5 Fig). TVB-3664 can block the *de novo* biosynthesis of fatty acids by inhibiting the activity of FASN, whereas triacsin C blocks the formation of acyl-CoA from fatty acids in the pathway of TAG synthesis by targeting ACSLs (Fig 8A). Interestingly, a non-cytotoxic concentration of TVB-3664 treatment did not affect the phosphorylation of AKT or the expression of KSHV lytic gene RTA in both WT and PINLYP KO cells (Fig 8B). In contrast, treatment of ACSL5 inhibitor triacsin C significantly decreased the phosphorylation of AKT and the expression of KSHV lytic gene RTA in PINLYP KO cells during Dox-induced KSHV reactivation, restoring the effect of PINLYP deficiency (Fig 8B). However, overexpression of ACSL5 alone had no effect on the expression of KSHV lytic gene RTA upon Dox-induced reactivation (S6 Fig), suggesting that its activity is also regulated through mechanisms other than transcriptional control. Lysophosphatidylcholine (LysoPC), a direct hydrolytic product of cPLA2α, is known to activate multiple signaling [40]. We then examined a possible link between pathways induced by LysoPC and KSHV lytic reactivation. We used the most common LysoPC (16:0, 18:0) to treat iSLK.RGB cells and found that treatment at 20 μM of LysoPC (16:0, 18:0) was toxic to the cells. Treatment at a non-toxic concentration of 5 μM did not affect the expression of the KSHV lytic gene RTA in iSLK.RGB cells (S7 Fig), suggesting that LysoPC has no effect on KSHV lytic reactivation.

Next, we investigated whether the elevated levels of TAG biosynthesis are responsible for the enhancement of AKT activation and KSHV lytic reactivation in PINLYP KO cells. There are two evolutionarily unrelated DGAT enzymes, DGAT1 and DGAT2, which catalyze nearly all TAG synthesis (Fig 8A). We used DGAT1 and DGAT2 specific inhibitors to treat WT and PINLYP KO cells upon Dox-induced KSHV lytic reactivation. The cytotoxicity of DGAT inhibitors was examined in WT and PINLYP KO (C4) cells before the treatment (S8A Fig). Treatment with a nontoxic concentration (50μM) of DGAT1-specific inhibitor significantly reduced the phosphorylation of AKT and the expression of KSHV lytic gene RTA in PINLYP KO cells, whereas a nontoxic concentration (50μM) of DGAT2-specific inhibitor showed a marginal impact on AKT phosphorylation and KSHV RTA expression (Fig 8C and 8D), suggesting that DGAT-mediated TAG synthesis driven by PINLYP deficiency is responsible for the enhancement of KSHV lytic reactivation in PINLYP KO cells. Furthermore, we used a nontoxic concentration (50μM) of DGAT inhibitors to treat KSHV-positive lymphoma B cell line BCBL1 and obtained the same phenotype as that in iSLK-RGB cells (S8A and S8B Fig). Taken together with the aforementioned data, our results demonstrate that PINLYP deficiency induces an uplift of TAG biosynthesis by increasing cPLA2α activity and ACSL5 expression, which remodels phospholipid metabolism and ultimately elevates the phosphorylation of AKT and KSHV lytic reactivation. More importantly, our experiments demonstrated that DGAT blockage effectively reduces KSHV lytic reactivation from latency, indicating that DGATs may serve as potential targets for inhibiting viral lytic reactivation.

## Discussion

In this study, we demonstrate that PINLYP plays an important role in remodeling phospholipid metabolism and TAG biosynthesis, mediating AKT activation and KSHV lytic reactivation from latency (Fig 8E). PINLYP deficiency in a KSHV latently infected iSLK.RGB cell pronouncedly elevates the replication of the viral genome, the expression of viral lytic genes, and the production of progeny viruses during KSHV lytic reactivation. Furthermore, PINLYP inhibits the activation of phospholipase cPLA2α which is one of the most important enzymes involved in phospholipid metabolism. We detected and quantified lipid and metabolite species in WT and PINLYP KO cells, and lipidomic profiling revealed that PINLYP deficiency predominantly increases TAG subclasses. Blocking the *de novo* synthesis of TAG by specific inhibitors triacsin C and DGAT1 suppresses AKT activation and KSHV lytic gene expression in PINLYP KO cells during viral reactivation, restoring the phenotype similar to that in WT cells. These results resolve the underlying mechanism how KSHV latent infection triggers the remodeling of phospholipid metabolism by utilizing the host cellular factor PINLYP, providing a new insight into the regulation of chronic herpesvirus infection by the host phospholipid reprogramming, particularly TAG biosynthesis.

PINLYP belongs to the Ly6/uPAR superfamily, whose expression can be induced or suppressed by viral infection. The Ly6/uPAR family members have been shown to interplay with viral pathogens and exhibit distinct functions in responses to different virus infections. The most extensively studied Ly6/uPAR protein LY6E enhances the infection of influenza A virus, flaviviruses, and human immunodeficiency virus 1 [41–43], but inhibits coronavirus infection by impairing viral fusion [44]. Another well documented Ly6/uPAR protein, CD59, is shown to involve KSHV and other herpesvirus infection [45,46]. Interesting, we observed that *de novo* KSHV infection of HUVECs induces PINLYP expression until the establishment of KSHV latent infection in the infected cells, indicating that PINLYP expression might be beneficial for KSHV latent infection. Consistently, in PINLYP KO iSLK.RGB cells prior to Dox induction, we even observed a small population of GFP-positive cells in which the lytic promoter of KSHV driving GFP expression is activated, representing KSHV-reactivating cells. Furthermore, the genome copies of KSHV and expression of viral latent and lytic genes were also markedly increased in PINLYP KO cells prior to Dox treatment, highlighting the important role of PINLYP in inhibiting KSHV lytic reactivation to facilitate viral latent infection. How KSHV *de novo* infection induces PINLYP expression remains to be resolved in a future study.

We have previously reported that PINLYP regulates type I IFN innate immunity and protects the host against virus infection [35]. IFN and IFN-inducible genes can control the viral latency of gammaherpesviruses KSHV and MHV68 by inhibiting lytic gene expression [5]. It’s reasonable to assume that PINLYP inhibits KSHV lytic reactivation via its function related to IFN signaling. However, no significant difference was observed for the phosphorylation level of IRF3 and STAT1 between WT and PINLYP KO iSLK.RGB cells after Dox treatment, indicating that PINLYP has no appreciable effect on the IFN signaling pathway during KSHV lytic reactivation, ruling out the possibility that IFN signaling is involved in PINLYP-mediated inhibition of KSHV lytic reactivation. Considering that PINLYP mainly contains a PLA2 inhibitor domain, cPLA2α is well documented to activate PI3K/AKT signaling [47], and AKT activation is required for KSHV lytic reactivation, it’s rational for us to verify whether PINLYP could mediate AKT activation and KSHV lytic reactivation by regulating cPLA2α activity. Our data on cPLA2α specific inhibitor pyrrophenone supports this hypothesis. It has been reported that pyrrophenone not only inhibits the activity of cPLA2α, it also can reduce cPLA2α expression level in some cell subtypes [48]. We also observed a decreased expression of cPLA2α and AKT in pyrrophenone-treated WT and PINLYP KO iSLK.RGB cells. Reduction of cPLA2α expression could inhibit phospholipid metabolism, which leads to a reduced inflammation response and cytokine production that can play a feedback role in regulating the expression of cPLA2α and AKT. However, whether PINLYP directly or indirectly inhibits the activity of cPLA2α remains to be further determined. Furthermore, even though we observed a significant effect of PINLYP on the level of p-AKT, only a slight decrease of p-mTOR level was observed in PINLIP KO cells upon KSHV lytic reactivation, which might attribute to the complexity between PI3K/AKT/mTOR signaling and lipid metabolism-associated mTOR signaling pathways.

The advancement of lipidomics has accelerated our understanding of viral infections in recent years. Viruses take advantage of host cell phospholipids by reprogramming the lipid metabolism of the cell. Previous studies have shown that human cytomegalovirus infection rewires the lipid metabolism of the host cells, upregulating fatty acid metabolism and providing the saturated very-long-chain fatty acids that are required for the production of infectious virion progeny [49–52]. Murine herpesvirus 68 (MHV68) infection also reprograms lipid metabolism, inducing a peak in TAG early during infection and an increase in free fatty acids and DAG later in the viral life cycle, whereas MHV68-driven induction of lipogenesis is essential for virion production [53]. KSHV latent infection also dramatically alters the lipid profile of endothelial cells [30]. However, the underlying mechanism of virus-driven lipid metabolism is unknown. We showed that KSHV hijacks the host factor PINLYP to reprogram phospholipid metabolism by regulating ACSL5 expression and TAG biosynthesis. Our result resolves the mechanism how virus like KSHV remodels the host lipid metabolism during latent infection.

To ensure the maintenance of cellular homeostasis, many lipid metabolic pathways are coordinated with pathways that involve amino acids, carbohydrates and many others. Conversely, dysregulated lipid homeostasis can also lead to a variety of complications by integrating amino acid and carbohydrate metabolism. PINLYP deficiency had a minimal impact on energy metabolism, however, it resulted in a decrease in expression levels of most genes related to amino acid and carbohydrate metabolic pathways (S9 Fig). This suggests that PINLYP deficiency enhances TAG biosynthesis while simultaneously reducing the metabolism of amino acids and carbohydrates in KSHV latently infected cells, highlighting the broad impact of PINLYP deficiency and its key roles in maintaining cellular homeostasis.

Phospholipids and their associated regulatory enzymes have been shown to interfere with viral infection, displaying proviral or antiviral function [54–57]. Lipid metabolites regulate the pathogenic phase of influenza virus infection [58]. PLSCR1 interacts with Epstein-Barr virus protein BZLF1 and represses BZLF1-mediated lytic gene transcription [59]. Our lipidomic data show an increase in accumulated TAG subclasses, an output of TAG biosynthesis in PINLYP KO iSLK.RGB cells during KSHV lytic reactivation, suggesting that PINLYP negatively regulates TAG biosynthesis. Blocking TAG biosynthesis by DGAT1 almost completely restores the phenotype of PINLYP deficiency, demonstrating that PINLYP inhibition of KSHV lytic reactivation is dependent on TAG biosynthesis. A key component in TAG synthesis is fatty acids, which can be supplied through lipid remodeling or *de novo* synthesis from acetyl-CoA catalyzed by the key enzyme FASN. TVB-3664, a pharmacological inhibitor of FASN, blocks fatty acid *de novo* synthesis but has a trivial impact on the phosphorylation of AKT and KSHV lytic reactivation, indicating that fatty acid *de novo* synthesis is not crucial for PINLYP regulation of KSHV lytic reactivation. Given that KSHV *de novo* infection of HUVECs induces PINLYP expression, our data highlight that KSHV hijacks the cellular factor PINLYP to remodel phospholipid metabolism, particularly TAG biosynthesis, in favor of its own latency. Thus, our study provides a new potential target for limiting viral lytic reactivation and treating associated diseases.

## Materials and methods

### Cell lines

The 293T and 293FT cells were cultured in DMEM (Gibco) supplemented with 10% heat-inactivated FBS (Gibco), 50 U/mL penicillin, and 50 μg/mL streptomycin at 37°C and 5% CO_2_. The iSLK-RGB-BAC16 (iSLK.RGB) cell line was established in the laboratory of Professor Jae U Jung (Learner Research Institute, Cleveland Clinic) [60]. KSHV-RGB-BAC16 containing red, green and blue reporter fluorescent protein is derived from KSHV-BAC16 by homologous recombination. Monomeric red fluorescent protein 1 (mRFP) controlled by constitutive promoter EF1-α, enhanced green fluorescent protein (EGFP) controlled by pPAN promoter, and monomeric blue fluorescent protein (tagBFP) controlled by pK8.1 promoter were introduced to mark the expression of latent, early, and late viral genes, respectively.

The iSLK.RGB PINLYP KO cell lines were generated from iSLK.RGB cell line by knocking out PINLYP gene using the CRISPR/Cas9 gene-editing technology. Four pairs of sgRNA (S2 Table) were selected and used. iSLK.RGB cells were transduced with lentiviruses packaged with lentiCRISPR-vec or lentiCRISPR-PINLYP-sgRNA. Limiting dilution cell culture was performed after 48 h post-transduction. Single cell clones C2 and C4 from two different pairs of sgRNAs were selected and the knockout efficiency of PINLYP was detected by qRT-PCR and western blot analyses, followed by sequencing verification.

The iSLK.RGB cell line and iSlK.RGB PINLYP KO cell lines were cultured in DMEM (Gibco) supplemented with 10% heat-inactivated FBS (Gibco), 50 U/mL penicillin, 50 μg/mL streptomycin, 1 μg/mL Puromycin, 100 μg/mL Hygromycin B, and 50 μg/mL G418 at 37°C and 5% CO_2_. Human primary endothelial cells (HUVEC) from Lonza were maintained in EGM-2 (Lonza, Basel, Switzerland) at 37°C and 5% CO2. BCBL1 cells were cultured in RPMI1640 (Gibco) supplemented with 10% heat-inactivated FBS (Gibco), 50 U/mL penicillin, and 50 μg/mL streptomycin at 37°C and 5% CO2.

### Plasmid construction

The primers used for gene cloning and plasmid construction are listed in S2 Table. PCR employed PrimeSTAR HS DNA Polymerase (R010A, TaKaRa) and the instrument for PCR was BIO-RAD T100 Thermal Cycler.

The lentiCRISPR-PINLYP-sg1/2/3/4 vector for gene knockout was constructed according to the method given in the paper published by Feng Zhang’s laboratory. Two primer oligos containing sgRNA sequences were first annealed and naturally cooled in a 95°C water bath. Then they were ligated with the lentiCRISPR-v2 vector recovered after digestion with BsmBI (R0739, NEB). All constructed plasmids were transformed into Stbl3 Competent *E. coli* and selected on antibiotic-containing (Ampicillin) luria broth (LB) agar plates. All plasmids were confirmed by sequencing.

The plasmid pGL2-RTAp-luciferase containing the promoter of KSHV switch protein RTA and the plasmid pCAGGS-LANA-SF expressing KSHV latent protein LANA were obtained as a gift from Professor Lan Ke (Wuhan University).

To generate PINLYP (pLVX-PINLYP-AU1-puro) and cPLA2α (pLVX-cPLA2α-Flag-puro) expression plasmids, the coding sequences were amplified from BCBL1 cell cDNA and subcloned into the pLVX-IRES-puro vector. The resulting constructs were transformed into Stbl3 Competent *E. coli* and selected on ampicillin-containing Luria-Bertani agar plates. Sequence verification was performed on all plasmid inserts to ensure integrity.

### Lentivirus packaging and transduction

psPAX2, pMD2.G, and target plasmid were transfected into 293FT cells to produce lentiviral particles. After that, the viral supernatant was collected every 12 h and replaced with fresh medium. After the last collection at 72 h. The viral supernatants were mixed, centrifuged, and frozen at -80°C. When the target cell density reached approximately 80%, cell culture medium was discarded, the lentivirus supernatant was added. Specifically, 2 mL of lentiviral supernatant was introduced to each well of a 6-well plate, followed by the addition of polybrene to reach a final concentration of 5 μg/mL. The plates were subsequently centrifuged at 2000 rpm for 2 h at room temperature. After 12 h post-transduction, the viral supernatant was removed and replaced with fresh medium.

### Western blotting

Cells were harvested, centrifuged at 5000 rpm for 5 min, and subsequently washed with PBS. The cell pellets were resuspended with 1x SDS Loading Buffer, sonicated three times using an ultrasonic breaker (VCX 130, Vibra cell) with 50% Ampl. Samples were boiled in a metal bath at 100°C for 5 min and placed on ice.

Western blot was carried out with standard method. Gel electrophoresis was started at 85 V for 40 min and 120 V for 60 min. Transfer conditions: 100 V, 90 min. After transfer on ice, the membranes were blocked with 5% skim milk for 1 h at room temperature and subsequently incubated with primary antibody overnight at 4°C. After wash three times with TBST, 5 min each time, the membrane was incubated with horseradish peroxidase-conjugated secondary antibody for 1 h at room temperature, followed by wash with TBST for three times, 5 min each time, and subjected to detection with chemiluminscent HRP substrate (P90720, Millipore). Antibodies used in this study were shown in S1 Table. All the reported Western blot data was representative of three experiments.

### RNA purification and qRT-PCR

Total RNA extraction was performed using TRIZOL reagent. cDNA was synthesized from using the ReverTra Ace qPCR RT Master Mix with gDNA Remover (FSQ-201, TOYOBO). KOD-SYBR qPCR Mix (QKD-201, TOYOBO) was used for qRT-PCR reactions. The specificity of amplification was verified by melting curve analysis. Ct values of target genes were normalized to GAPDH and relative gene expression levels between conditions were calculated via the 2-ΔΔCt method. All primers used for qRT-PCR analyses were listed in S2 Table. qRT-PCR reactions were performed using a Real-time PCR machine (LightCycler 96, Roche). Each experiment was performed in three replicates and repeated three times.

### Relative quantification of KSHV genomic copy number

The TIANamp Genomic DNA Kit (DP304-3, TIANGEN) was utilized for the extraction of genomic DNA from the collected cells, followed by qPCR for relative quantification. The abundance of the KSHV genome was determined by TR and K9 coding regions, while the abundance of the GAPDH genome sequence was utilized as an internal reference for normalization. All primers used for relative quantification of KSHV genomes are listed in S2 Table.

### Cell transfection

3.5x10^5^ iSLK.RGB cells were seeded into each well of 6-well plates the day before transfection, the medium supernatant was changed to serum-free DMEM 2h before transfection, 2 μg of plasmid was transfected into the cells with FuGENE HD Transfection Reagent (E2311, Promega). After 1 h, 200 μL FBS was supplemented.

1x10^6^ 293T cells were seeded into each well of 6-well plates the day before transfection. 2 μg of plasmid was transfected into 293T cells with Neofect DNA transfection reagent (TF201201, Neofect biotech Co.,Ltd).

### KSHV infection of 293T and HUVEC cells

293T cells were seeded the day before infection and the cell density was approximately 70% at the time of infection. Culture supernatant containing KSHV viral particles was collected and centrifuged at 4000 rpm for 10 min to remove cell debris. The 293T cell culture medium was discarded, and 200 μL virus supernatant (12-well plate) was added, followed by the addition of polybrene to reach a final concentration of 5 μg/mL. The cell culture plate was subjected to intermittent shaking every 15 minutes to ensure uniform distribution of the viral supernatant across the plate surface. After 1 h, 1 mL of fresh medium was supplemented. HUVEC cells were infected with KSHV at MOI = 1 in the presence of 8 μg/mL polybrene (Millipore).

### Dual luciferase reporter assay

Dual luciferase reporter assay was performed using Dual-Lumi II Luciferase Reporter Gene Assay Kit (Beyotime) according to the manufacturer’s instructions. Cells were transfected with luciferase reporter plasmid, protein-expressing plasmids, and Renilla plasmid which served as an internal control. After 48 h, cell lysates were harvested for dual luciferase detection using a luminometer (Bio Tek). Relative luciferase activity was determined by dividing the luminescence of firefly luciferase by the luminescence of Renilla luciferase.

### Cell viability assay

Cell viability was analyzed using a WST-8 cell counting kit-8 (Beyotime) according to the manufacturer’s instruction. In brief, cells were seeded onto 96-well plates at a density of 1 × 10^4^ cells per well. The next day, cells were treated with a series concentration of AKT, FASN, ACSL5, or DGAT inhibitors for 24 h. CCK-8 solution was added to each well and the cultures were incubated at 37°C, 5% CO2 for 2 h. The absorbance at a wavelength of 450 nm was determined using a Synergy H1 microplate reader (Bio Tek). Each sample had three replicates and the experiment was done twice.

### RNA-seq

RNA-seq and data analysis were performed byBGI Genomics Co., Ltd. WT group included two iSLK.RGB.WT samples and KO group included two individual clones iSLK.RGB. KO C2 and C4. Briefly, total RNA of cells was extracted and mRNA was enriched by oligo-dT magnetic to construct RNAseq libraries. Quantity and quality of total RNA and final libraries was determined by Agilent 2100 before sequencing on BGISEQ-500. Reads were cleaned by SOAPnuke (BGI) and mapped to human genome by HISAT. Mapped reads were quantified using Bowtie2 and RESM. Differential expression analysis was performed by DEGseq. Gene Ontology and pathway enrichment were analyzed by function phyper.

### Lipid extraction

Total lipids were extracted by MTBE protocol [61]. Briefly, cell pellets were resuspended in 100 μL of water, followed by 360 µL of MeOH, 1.2 mL of MTBE and a mixture of internal standards (200 μmol of C17 ceramide, 200 μmol of C8 ceramide-1-phosphate, 200 μmol of cardiolipin14:0, 200 μmol of C6 glucosylceramide, 200 μmol of LPC17:0, 200 μmol of LPE13:0, 200 μmol of LPS17:1, 200 μmol of PA10:0, 1000 μmol of DLPC, 200 μmol of d_31_-PE31:1(16:0/18:1), 200 μmol of d_31_-PG31:1(16:0/18:1), 200 μmol of d_31_-PS31:1(16:0/18:1), 200 μmol of SM12:0, 200 μmol of d_5_-TAG(15:0/18:1/15:0)). Samples were vigorously vortexed at maximum speed for 10 minutes at 4°C and incubated for 1 h on a shaker at room temperature. Phase separation was induced by addition of 200 μL of water and incubation for 10 minutes. Samples were centrifugated at 1000 g for 10 minutes and the upper phase was transferred into 13 mm glass vials. To the lower phase, 400 μL of MTBE/MeOH/H_2_O mixture (10:3:1.5, v/v) was added and re-extracted. The combined upper phase was dried under nitrogen flow at room temperature. The dried samples were kept at -80 °C.

### Targeted lipidomics

Lipids were analyzed following previous protocols with slight modification [62]. Each sample was resuspended in 100 µL of CHCl_3_/MeOH mixture (1:1, v/v) and transferred to a glass vial with insert, 10 μL was used for injection. Lipidomic analyses were carried out on a UHPLC (Vanquish Flex) coupled with a quadrupole-Orbitrap mass spectrometer (Q Exactive Plus, Thermo Fisher Scientific), equipped with an Accucore column (C30, 250 x 3 mm, 2.6 μm, Thermo Fisher Scientific). Lipids were separated under a binary solvent system: mobile phase A was water containing 0.1% formic acid and 5 mM ammonium formate, mobile phase B was acetonitrile/isopropanol (1:2, v/v) containing 0.1% formic acid and 5 mM ammonium formate. Samples were eluted at a flow rate of 0.35 mL/min at 40 °C with the following gradient: 0–2 min, 5% B; 2–6 min, 5–70% B; 6–16 min, 70–100% B; 16–24 min, 100% B; 24–28 min, 5% B. The mass spectrometer was operated using a heated electrospray ionization (HESI) source (sheath gas flow rate: 51, aux gas flow rate: 13, spray voltage = -3.5 kV (-) or 3.9 kV (+), capillary temperature = 410 °C, S-lens RF level = 50.0, aux gas heater temperature = 320 °C) in both positive and negative mode using full scan analysis (m/z: 200–1700 resolution: 70,000, AGC target: 3e6, maximum IT: 100 ms) and TopN method (resolution: 17,500, AGC target: 1e5, maximum IT: 50 ms, (N)CE: 20, 25, 30). Lipids were quantified by extracting peak areas using TraceFinder (Thermo Fisher Scientific), converted into pmol following internal standard calibration curves, and normalized by the number of cells.

### Quantification and statistical analysis

Data were analyzed using GraphPad Prism 6, and student unpaired *t*-test was used to determine whether there were significant differences between samples (*p < 0.05, **p < 0.01, ***p < 0.001, ****p < 0.0001). Fold change was calculated as the ratio of means of two groups. *P* value (adjusted) was calibrated using Benjamini-Hochberg method. Fold change and −log_10_ [P] values are presented in the volcano plot, and points with Fold change > 2 and *P* < 0.05 were considered to be statistically significant. Principal component analysis (PCA) was calculated and shown using stats v.4.3.2. Heat map was drawn using pheatmap (v.1.0.12). Statistical analyses were performed using R (v.4.3.3).

## Supporting information

S1 Table Key resources and reagents. (DOCX)

S2 Table Primers. (DOCX)

S1 Fig PINLYP deficiency promotes KSHV lytic reactivation from latency. (A) Western blot and qRT-PCR detection of PINLYP knockout efficiency. (B) WT and PINLYP knockout (C2 and C4) iSLK-RGB cells were treated with Dox (1 μg/mL) for 48 h, mock control was WT cells treated with DMSO for 48h, supernatant was harvested to infect 293T cells and subsequently subjected to fluorescence imaging at 72 h post-infection. Image magnification: 10 × . (C) Quantitative analyses of RFP-positive KSHV-infected 293T cells by FACS analyses for (B). Error bars indicated SEM, *p < 0.05, **p < 0.01, ***p < 0.001, two-tailed unpaired *t*-test.
(TIF)

S2 Fig PINLYP has no effect on the activation of KSHV RTA promoter. 293T cells were transfected with RTA promoter luciferase plasmid as well as renilla reporter as an internal control, co-transfected with PINLYP-AU1 and/or LANA-Flag expression plasmids as indicated. Luciferase activity was measured and normalized to renilla activity. PINLYP and LANA expression were detected by immunoblotting with the AU1 and Flag antibodies, respectively. The error bars indicated SEM, two-tailed unpaired *t*-test, ns represents no significant.
(TIF)

S3 Fig PINLYP deficiency increases AKT phosphorylation during KSHV lytic reactivation. (A) WT vs PINLYP KO iSLK-RGB cells were treated with Dox (1 μg/mL) for 24 h and subjected to RNA-seq analyses, MA plot showed Up-regulated and Down-regulated genes for WT vs KO cells. (B) Quantitative analyses of p-AKT for Fig 4B. (C) Quantitative analyses of p-AKT and RTA for Fig 4D. (D) Wild-type (WT) and PINLYP KO (C4) iSLK-RGB cells were treated with an indicated concentration of AKT inhibitor AKT-KI for 24 h, following by cytotoxicity detection using CKK. Three replicates for each concentration of every sample. IC50 was calculated and indicated. (E) WT vs PINLYP KO iSLK-RGB cells were treated with Dox (1 μg/mL) for 24 h and subjected to immunoblot analyses with the specific antibodies as indicated.
(TIF)

S4 Fig PINLYP deficiency remodels phospholipid metabolism during KSHV lytic reactivation. WT vs PINLYP KO iSLK-RGB cells were subjected to lipidomic analyses. Plots showed comparative statistical analyses of lipid subclasses. Unpaired *t*-test data are graphed on the y axis as fold change relative to WT cells. All error bars indicated SEM, *p < 0.05, **p < 0.01, ***p < 0.001, ****p < 0.0001, ns represents no significant. AcylCer: 1-O-acylceramides, Cer: Ceramides, PC: Phosphatidyl choline, PE: Phosphatidyl ethanolamine, PS: Phosphatidyl serine, LPC: Lyso-phosphatidyl choline, LPE: Lyso-phosphatidyl ethanolamine, LPS: Lyso-phosphatidyl serine, CerP: Ceramides phosphate, HexCer: Hexosylceramides, CL: Cardiolipin, PA: Phosphatidic acid, PG: Phosphatidyl glycerol, SM: Sphingomyelin.
(TIF)

S5 Fig Cytotoxicity of FASN inhibitor TVB-3664 and ACSL5 inhibitor Triacsin C. Wild-type (WT) and PINLYP KO (C4) iSLK-RGB were treated with an indicated concentration of FASN inhibitor TVB 3664 or ACSL5 inhibitor Triacsin C for 24 h, following by cytotoxicity detection using CKK. Three replicates for each concentration of every sample. IC50 was calculated and indicated.
(TIF)

S6 Fig ACSL5 overexpression alone is not sufficient to regulate KSHV lytic gene expression. iSLK-RGB cells were transfected with a vector (Vec) or a ACSL5-expressing plasmid with a C-terminal His tag using the indicated reagents. At 24 h post-transfection, the cells were stimulated with Dox (1 μg/mL) for 24 h and subsequently subjected to immunoblot analyses with the indicated antibodies.
(TIF)

S7 Fig Lyso-PC has no effect on KSHV lytic reactivation. iSLK-RGB cells were treated with Dox (1 μg/mL), along with Lyso PC (16:0), Lyso PC (16:0) or Lyso PC (18:0) for the indicated time, followed by immunoblot analyses with RTA and GAPDH antibodies.
(TIF)

S8 Fig DGAT inhibitor suppressed TPA-induced RTA expression in BCBL1 cells. (A) Wild-type (WT), PINLYP KO (C4) iSLK-RGB, or BCBL1 cells were treated with an indicated concentration of DGAT1 inhibitor DGAT1 I or DGAT2 inhibitor DGAT2 I for 24 h, following by cytotoxicity detection using CKK. Three replicates for each concentration of every sample. IC50 was calculated and indicated. (B) BCBL1 was treated with TPA (20 ng/mL) in the presence or absence of DGAT inhibitor DGAT1 I (50 μM) or DGAT2 I (50 μM) for 24 h, followed by immunoblot analyses with the specific antibodies as indicated.
(TIF)

S9 Fig PINLYP deficiency alters amino acid and carbohydrate metabolism. Heat map depicted the enrichment analyses of differentially expressed genes related to amino acid, carbohydrate, and energy metabolism pathways for RNA-seq data from WT and PINLYP KO iSLK-RGB cells.
(TIF)
