# Supplementary material for: PINLYP-mediated phospholipid metabolism reprogramming contributes to chronic herpesvirus infection
Source: PLoS Pathog. 2025 May 15;21(5):e1013146. doi: 10.1371/journal.ppat.1013146 (PMC12080810; doi:10.1371/journal.ppat.1013146)
Supplement: S1 Table — (DOCX) [file ppat.1013146.s001.docx]

**S1 Table. Key resources and reagents**

|  | | |
| --- | --- | --- |
| **REAGENT or RESOURCE** | **SOURCE** | **IDENTIFIER** |
| **Antibodies** | | |
| GAPDH | Santa Cruz Biotechonogy | sc-32233; RRID: AB_627679 |
| mPINLYP | This study | N/A |
| hPINLYP | This study | N/A |
| LANA | Prof. Qiliang Cai 's Lab, Fudan University | N/A |
| RTA | Prof. Ke Lan 's Lab, Wuhan University | N/A |
| ORF45 | Prof. Ersheng Kuang's Lab, Sun Yat-sen University | N/A |
| Anti-KSHV K8 alpha antibody | Abcam | ab36617-100; RRID: AB_775935 |
| Purified anti-AU1 Epitope Tag | BioLegend | 901901; RRID:AB_2565013 |
| Mouse Anti-FLAG Monoclonal Antibody, HRP Conjugated | Sigma | A8592-1MG; RRID:AB_439702 |
| IRF3 | Cell Signaling Technology | 4302S;RRID:AB_1904036 |
| Phospho-IRF-3 (Ser396) | Cell Signaling Technology | 29047S;RRID:AB_2773013 |
| STAT1 | Cell Signaling Technology | #9172;RRID:AB_2198300 |
| Phospho-STAT1 (Tyr701) | Cell Signaling Technology | 7649P;RRID:AB_10950970 |
| AKT | Cell Signaling Technology | 4685S;RRID:AB_2225340 |
| Phospho-AKT (Ser473) | Cell Signaling Technology | 4058S;RRID:AB_331168 |
| Phospho-AKT (Thr308) | Cell Signaling Technology | 4056S;RRID:AB_331163 |
| mTOR | Cell Signaling Technology | #2972;RRID:AB_330978 |
| Phospho-mTOR (Ser2448) | Cell Signaling Technology | 2971S;RRID:AB_330970 |
| PTEN | Cell Signaling Technology | 9188T;RRID:AB_2253290 |
| Phospho-p-PTEN (Ser380) | Cell Signaling Technology | #9551;RRID:AB_331407 |
| PI3 Kinase p110α | Cell Signaling Technology | #4255;RRID:AB_659888 |
| Phospho-PDK1 (Ser241) | Cell Signaling Technology | #3061;RRID:AB_2161919 |
| cPLA2 | Cell Signaling Technology | 5249S;RRID:AB_10622026 |
| Phospho-cPLA2 (Ser505) | Cell Signaling Technology | 53044S;RRID:AB_2799425 |
| PP2A-C | Cell Signaling Technology | 2259T;RRID:AB_561239 |
| HRP-conjugated 6*His, His-Tag Monoclonal antibody | Proteintech | HRP-66005; RRID:AB_2857904 |
| **Chemicals, peptides, and recombinant proteins** | | |
| Doxycycline | Sigma-Aldrich | D3072 |
| Pyrrophenone | Cayman | 13294 |
| Akt1/2 inhibitor | Sigma-Aldrich | A6730 |
| PrimeSTAR HS DNA Polymerase | TaKaRa | R010A |
| 50×TAE buffer | Sangon Biotech | B548101-0500 |
| Restriction Endonucleases | NEB | N/A |
| T4 DNA Ligase | NEB | m0202 |
| FuGENE HD Transfection Reagent | Promega | E2311 |
| NEOFECT^TM^ DNA transfection reagent | NEOFECT | TF201201 |
| Tenfect^TM^ DNA transfection reagent | TEYE | FT19301 |
| Lipofectamine^TM^ 2000 reagent | Invitrogen | 11668019 |
| Immbilon Western Chemiluminscent HRP Substrate | Millipore | P90720 |
| TRIZOL reagent | Invitrogen | 15596026 |
| Triacsin C | Abcam | AB141888 |
| TVB-3664 | MCE | HY-120062 |
| A922500 | Sigma-Aldrich | A1737 |
| PF-06424439 | Sigma-Aldrich | PZ0233 |
| Methyl Alcohol | Adamas | 75851G |
| Tert-Butyl Methyl Ether | Adamas | 28130H |
| CHCl_3_ | SCRC | 40064966 |
| Formic Acid | Adamas | 73553L |
| Ammonium Formate | CNW | CAEQ-4-014568-0050 |
| Acetonitrile | Merck | 1.00030 |
| Isopropanol | Merck | 1.01040 |
| DMSO | Yeasen | 67-68-5 |
| C17 Ceramide | Avanti | 860517P |
| C8 Ceramide-1-Phosphate | Avanti | 860532P |
| 14:0 Cardiolipin | Avanti | 750332P |
| C6 Glucosylceramide | Cayman | 24474 |
| 17:0 Lyso PC | Cayman | 33331 |
| 13:0 Lyso PE | Avanti | 856706P |
| 17:1 Lyso PS | Avanti | 858141P |
| 10:0 PA | Avanti | 830843P |
| DLPC | Avanti | 850335P |
| 16:0-d31-18:1 PE | Avanti | 860374P |
| 16:0-d31-18:1 PG | Avanti | 860384P |
| 16:0-d31-18:1 PS | Avanti | 860403P |
| 12:0 SM | Avanti | 860583P |
| 15:0-18:1-15:0 D5 TG | Avanti | 860901P |
| **Critical commercial assays** | | |
| DNA gel purification kit | TIANGEN | DP209 |
| Ordinary DNA purification kit | TIANGEN | DP204 |
| TIANprep Mini Plasmid Kit | TIANGEN | DP103 |
| NuleoBond Xtra Midi Kit | MACHEREY-NAGEL | 740410 |
| Ingenio^®^ Electroporation Kits and Solution | Miru | MIR 50115 |
| 10% ExpressCast PAGE Gel Preparation kit | NCM Biotech | P2012 |
| ReverTra Ace qPCR RT Master Mix with gDNA Remover | TOYOBO | FSQ-201 |
| KOD-SYBR qPCR Mix | TOYOBO | QKD-201 |
| TIANamp Genomic DNA Kit | TIANGEN | DP304 |
| Luciferase Reporter Gene Assay Kit | Beyotime | RG089M |
| Cell Counting Kit-8 | Beyotime | C0039 |
| **Recombinant DNA** | | |
| lentiCRISPR-PINLYP-sg1/2/3/4 | This study | N/A |
| pGL2-RTAp-luciferase | Prof. Ke Lan 's Lab, Wuhan University | N/A |
| pCAGGS-LANA-SF | Prof. Ke Lan 's Lab, Wuhan University | N/A |
| pLVX-PINLYP-AU1-puro | This study | N/A |
| pLVX-cPLA2α-flag-puro | This study | N/A |
| **Software** | | |
| GraphPad Prism 6 | GraphPad Software Inc. | https://www.graphpad.com/ |
| FlowJo | Tree Star Inc | https://www.flowjo.com |
| ImageJ | NIH | https://imagej.nih.gov/ij/index.html |
| **Other** | | |
| Gel Imaging System | Tanon | Tanon 2500 |
| 96-well PCR thermal cycler | BIO-RAD | T100 |
| Basic Power Supply | Beijing JUNYI Electrophoresis Co. | JY300 |
| Ultrasonic processors | Vibra-Cell | VCX 130 |
| Chemiluminescence Imaging System | Tanon | Tanon 5500 |
| Nucleofector^®^ 2b Device | Lonza | AAB-1001 |
| LightCycler 480 Multiwell Plate 96 | Roche | 4729692001 |
| Real-time PCR system | Roche | LightCycler^®^96 |
| Microplate Reader | Bio Tek | Synergy H1 |
| Leica Inverted Laboratory Microscope | Leica | Leica DM IL LED |
| Celesta flow cytometer | BD Biosciences | N/A |
|  | | |
