# Supplementary material for: PINLYP-mediated phospholipid metabolism reprogramming contributes to chronic herpesvirus infection
Source: PLoS Pathog. 2025 May 15;21(5):e1013146. doi: 10.1371/journal.ppat.1013146 (PMC12080810; doi:10.1371/journal.ppat.1013146)
Supplement: S2 Table — (DOCX) [file ppat.1013146.s002.docx]

**S2 Table. Primers**
