# Supplementary figures and images for: PINLYP-mediated phospholipid metabolism reprogramming contributes to chronic herpesvirus infection
Source: PLoS Pathog. 2025 May 15;21(5):e1013146. doi: 10.1371/journal.ppat.1013146 (PMC12080810; doi:10.1371/journal.ppat.1013146)

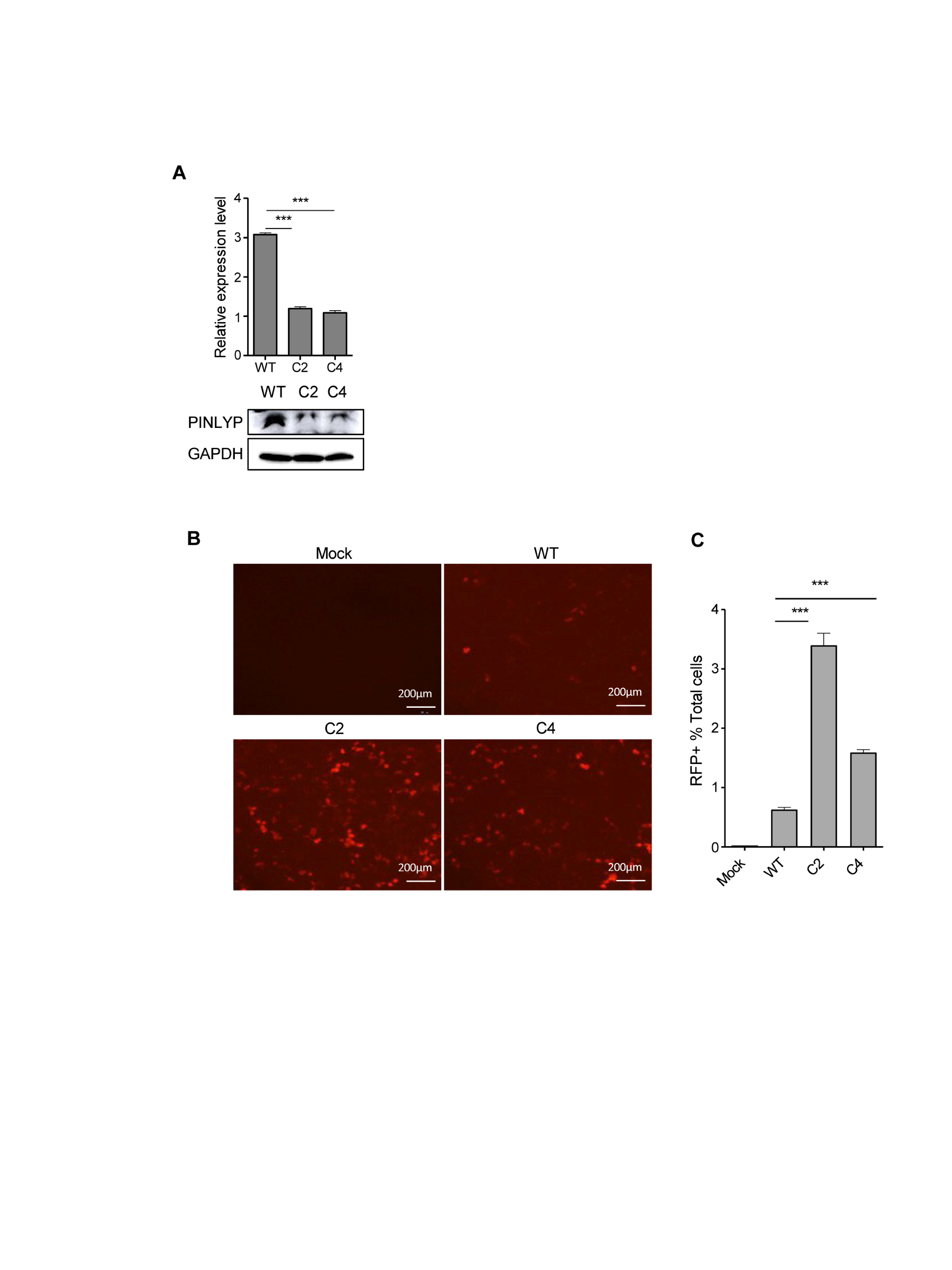

Supplement: S1 Fig — (A) Western blot and qRT-PCR detection of PINLYP knockout efficiency. (B) WT and PINLYP knockout (C2 and C4) iSLK-RGB cells were treated with Dox (1 μg/mL) for 48 h, mock control was WT cells treated with DMSO for 48h, supernatant was harvested to infect 293T cells and subsequently subjected to fluorescence imaging at 72 h post-infection. Image magnification: 10 × . (C) Quantitative analyses of RFP-positive KSHV-infected 293T cells by FACS analyses for (B). Error bars indicated SEM, *p < 0.05, **p < 0.01, ***p < 0.001, two-tailed unpaired t-test. (TIF) [file ppat.1013146.s003.tif]

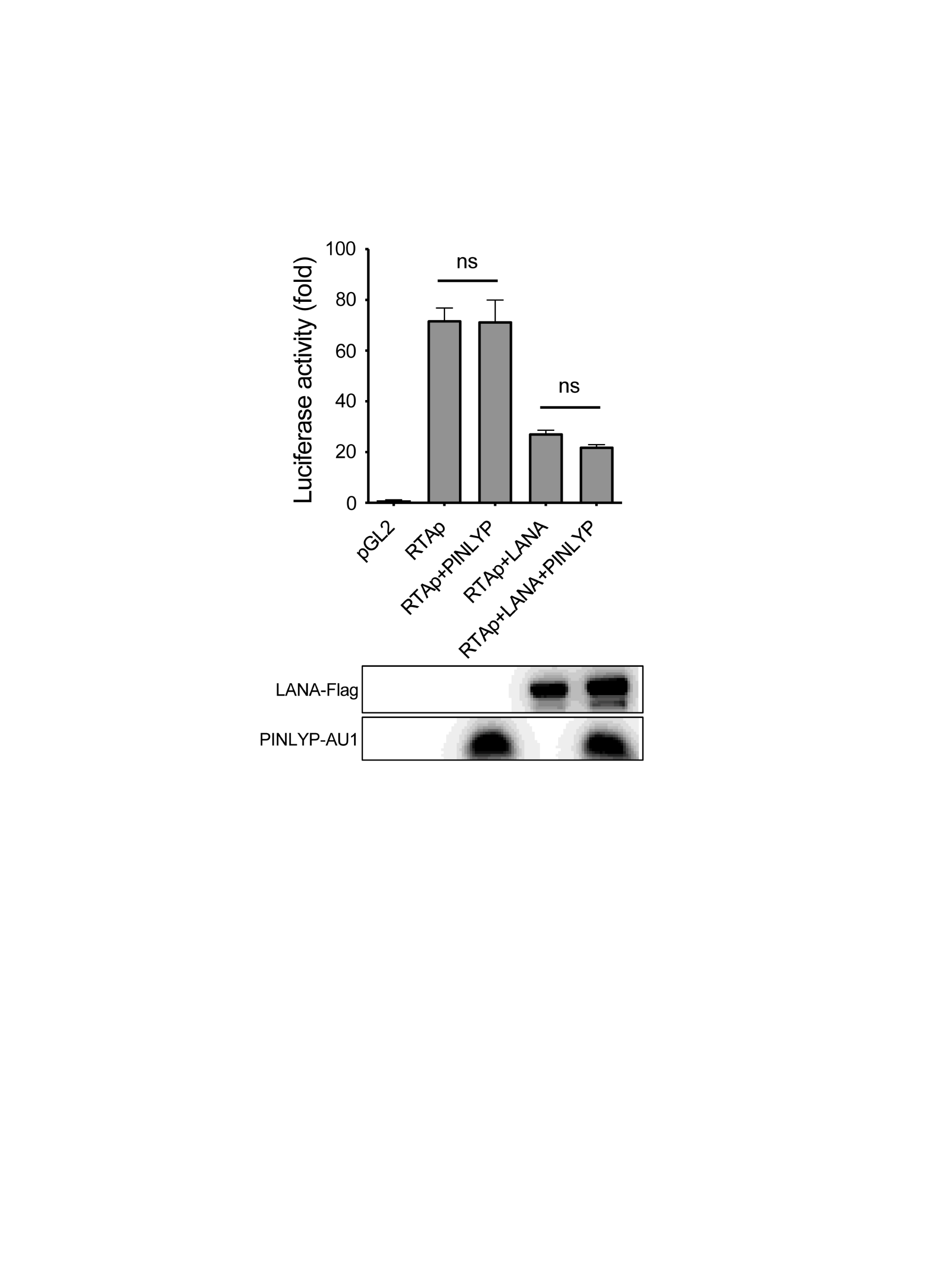

Supplement: S2 Fig — 293T cells were transfected with RTA promoter luciferase plasmid as well as renilla reporter as an internal control, co-transfected with PINLYP-AU1 and/or LANA-Flag expression plasmids as indicated. Luciferase activity was measured and normalized to renilla activity. PINLYP and LANA expression were detected by immunoblotting with the AU1 and Flag antibodies, respectively. The error bars indicated SEM, two-tailed unpaired t-test, ns represents no significant. (TIF) [file ppat.1013146.s004.tif]

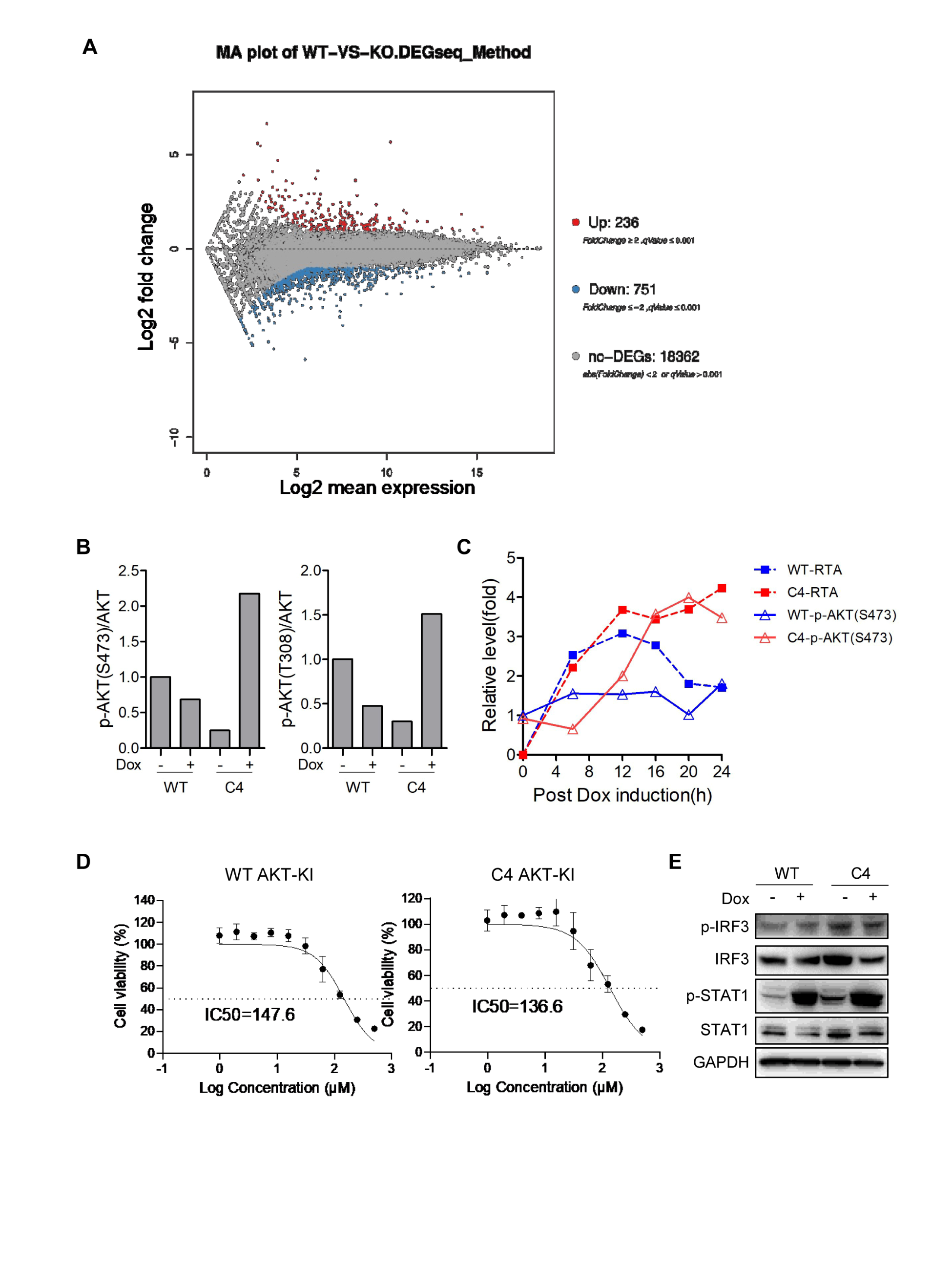

Supplement: S3 Fig — (A) WT vs PINLYP KO iSLK-RGB cells were treated with Dox (1 μg/mL) for 24 h and subjected to RNA-seq analyses, MA plot showed Up-regulated and Down-regulated genes for WT vs KO cells. (B) Quantitative analyses of p-AKT for Fig 4B. (C) Quantitative analyses of p-AKT and RTA for Fig 4D. (D) Wild-type (WT) and PINLYP KO (C4) iSLK-RGB cells were treated with an indicated concentration of AKT inhibitor AKT-KI for 24 h, following by cytotoxicity detection using CKK. Three replicates for each concentration of every sample. IC50 was calculated and indicated. (E) WT vs PINLYP KO iSLK-RGB cells were treated with Dox (1 μg/mL) for 24 h and subjected to immunoblot analyses with the specific antibodies as indicated. (TIF) [file ppat.1013146.s005.tif]

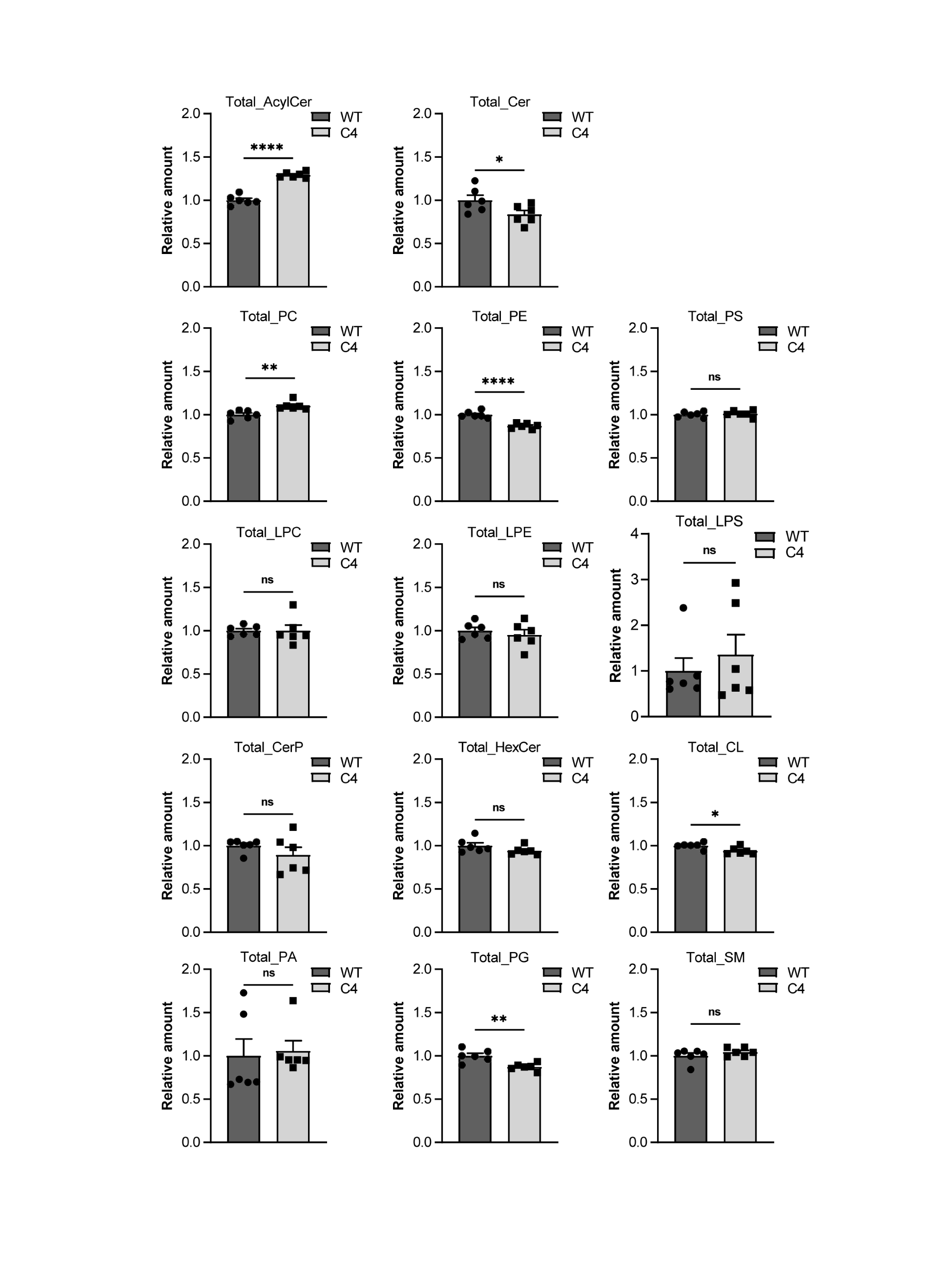

Supplement: S4 Fig — WT vs PINLYP KO iSLK-RGB cells were subjected to lipidomic analyses. Plots showed comparative statistical analyses of lipid subclasses. Unpaired t-test data are graphed on the y axis as fold change relative to WT cells. All error bars indicated SEM, *p < 0.05, **p < 0.01, ***p < 0.001, ****p < 0.0001, ns represents no significant. AcylCer: 1-O-acylceramides, Cer: Ceramides, PC: Phosphatidyl choline, PE: Phosphatidyl ethanolamine, PS: Phosphatidyl serine, LPC: Lyso-phosphatidyl choline, LPE: Lyso-phosphatidyl ethanolamine, LPS: Lyso-phosphatidyl serine, CerP: Ceramides phosphate, HexCer: Hexosylceramides, CL: Cardiolipin, PA: Phosphatidic acid, PG: Phosphatidyl glycerol, SM: Sphingomyelin. (TIF) [file ppat.1013146.s006.tif]

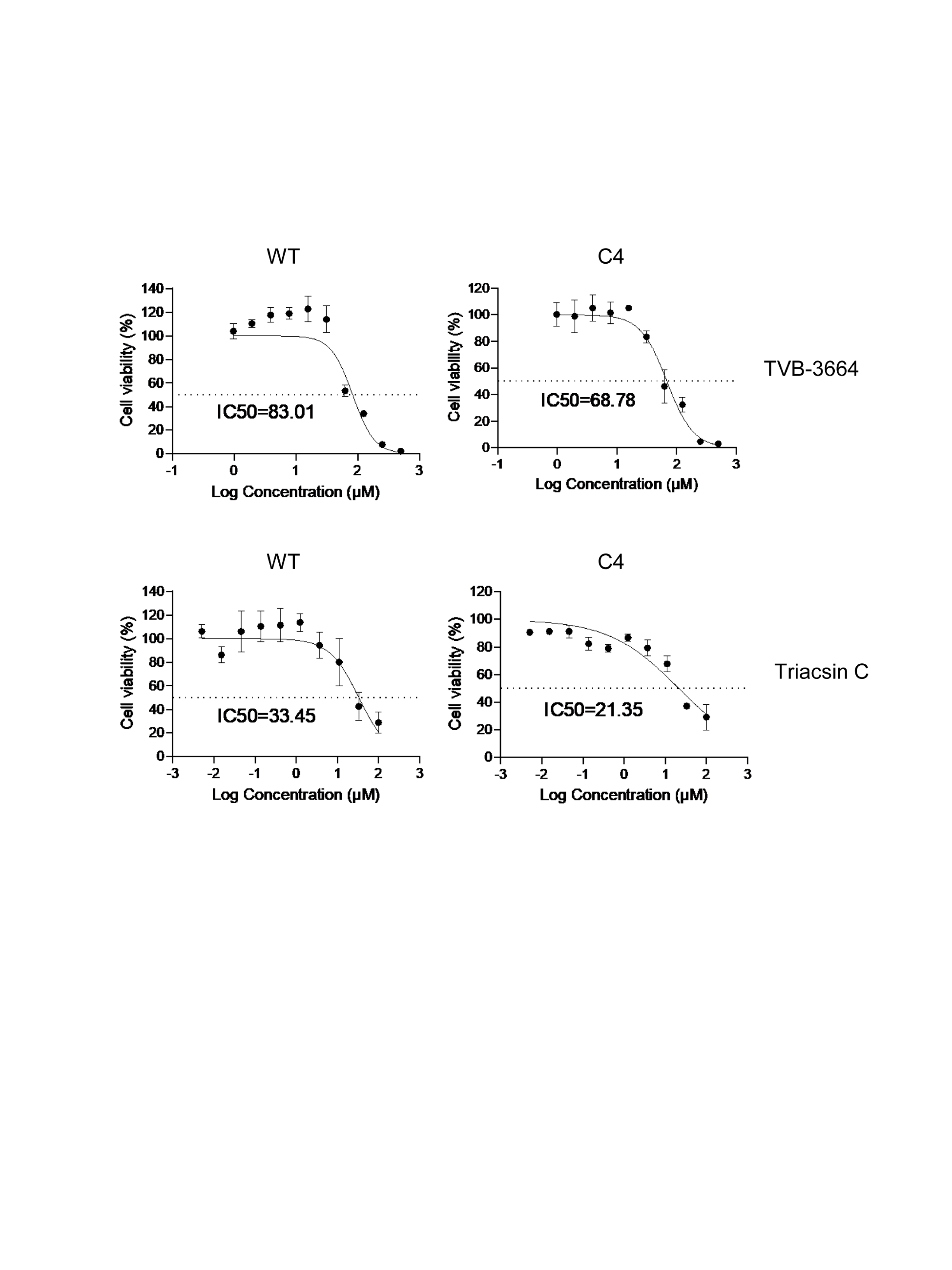

Supplement: S5 Fig — Wild-type (WT) and PINLYP KO (C4) iSLK-RGB were treated with an indicated concentration of FASN inhibitor TVB 3664 or ACSL5 inhibitor Triacsin C for 24 h, following by cytotoxicity detection using CKK. Three replicates for each concentration of every sample. IC50 was calculated and indicated. (TIF) [file ppat.1013146.s007.tif]

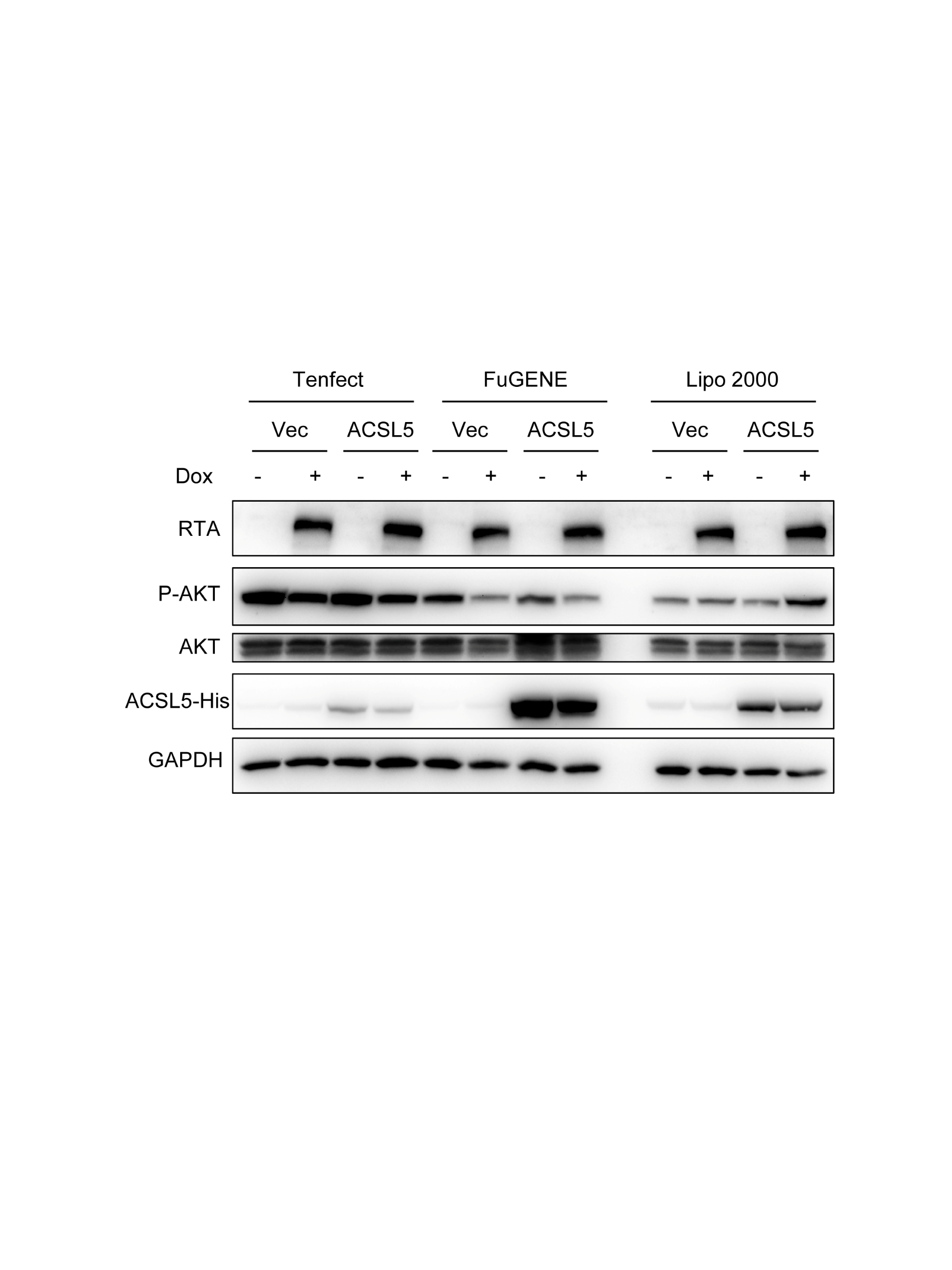

Supplement: S6 Fig — iSLK-RGB cells were transfected with a vector (Vec) or a ACSL5-expressing plasmid with a C-terminal His tag using the indicated reagents. At 24 h post-transfection, the cells were stimulated with Dox (1 μg/mL) for 24 h and subsequently subjected to immunoblot analyses with the indicated antibodies. (TIF) [file ppat.1013146.s008.tif]

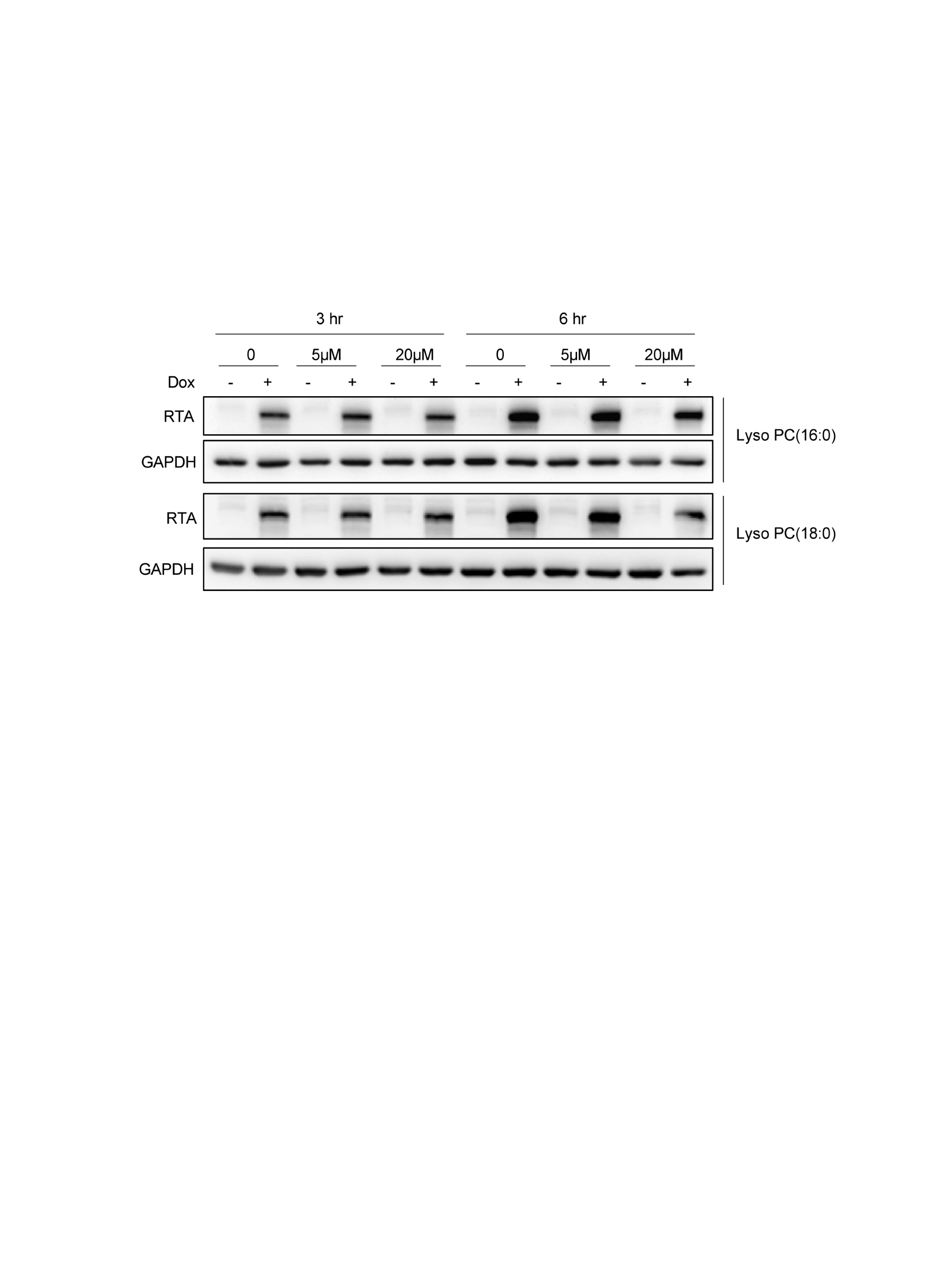

Supplement: S7 Fig — iSLK-RGB cells were treated with Dox (1 μg/mL), along with Lyso PC (16:0), Lyso PC (16:0) or Lyso PC (18:0) for the indicated time, followed by immunoblot analyses with RTA and GAPDH antibodies. (TIF) [file ppat.1013146.s009.tif]

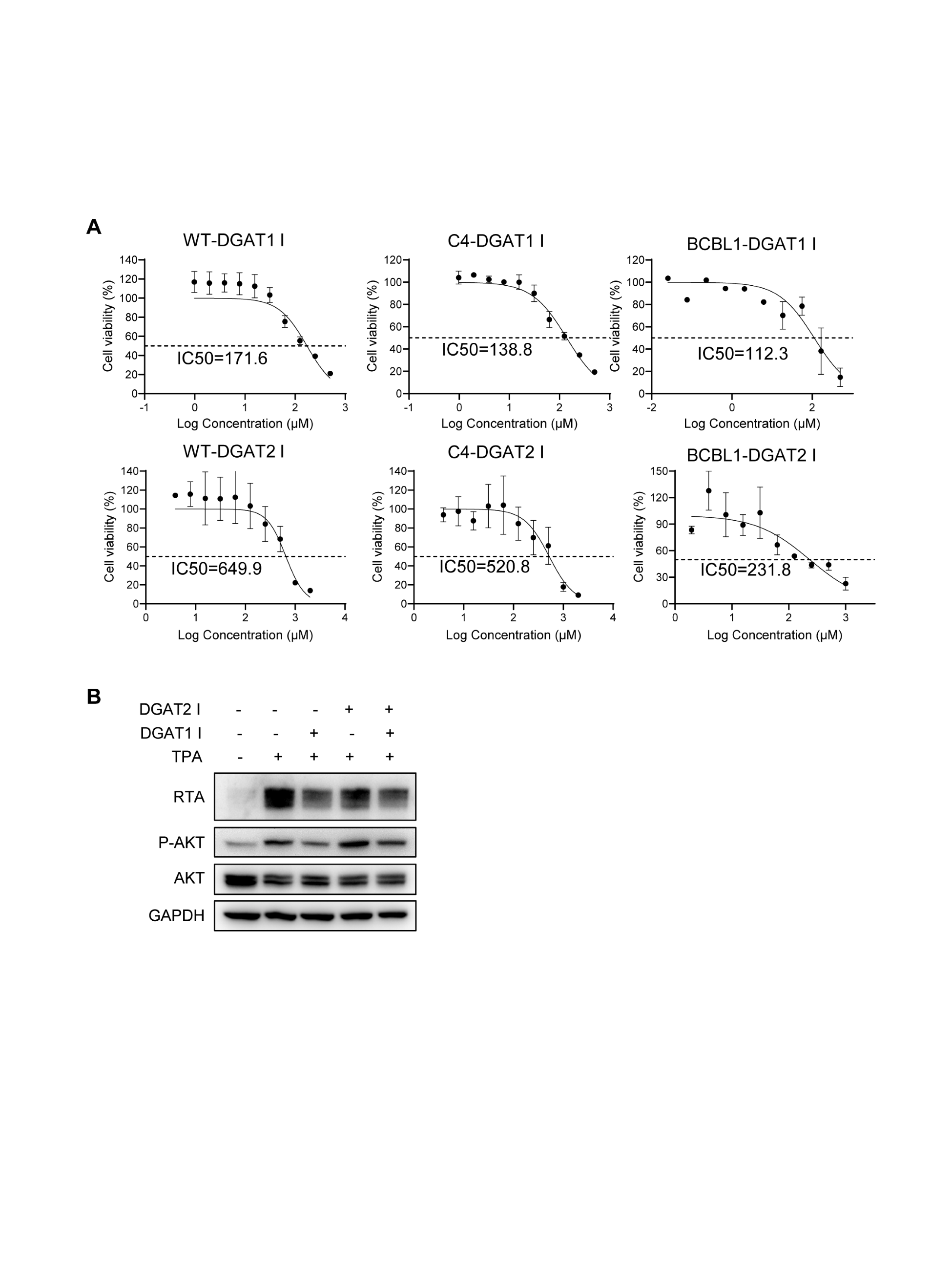

Supplement: S8 Fig — (A) Wild-type (WT), PINLYP KO (C4) iSLK-RGB, or BCBL1 cells were treated with an indicated concentration of DGAT1 inhibitor DGAT1 I or DGAT2 inhibitor DGAT2 I for 24 h, following by cytotoxicity detection using CKK. Three replicates for each concentration of every sample. IC50 was calculated and indicated. (B) BCBL1 was treated with TPA (20 ng/mL) in the presence or absence of DGAT inhibitor DGAT1 I (50 μM) or DGAT2 I (50 μM) for 24 h, followed by immunoblot analyses with the specific antibodies as indicated. (TIF) [file ppat.1013146.s010.tif]

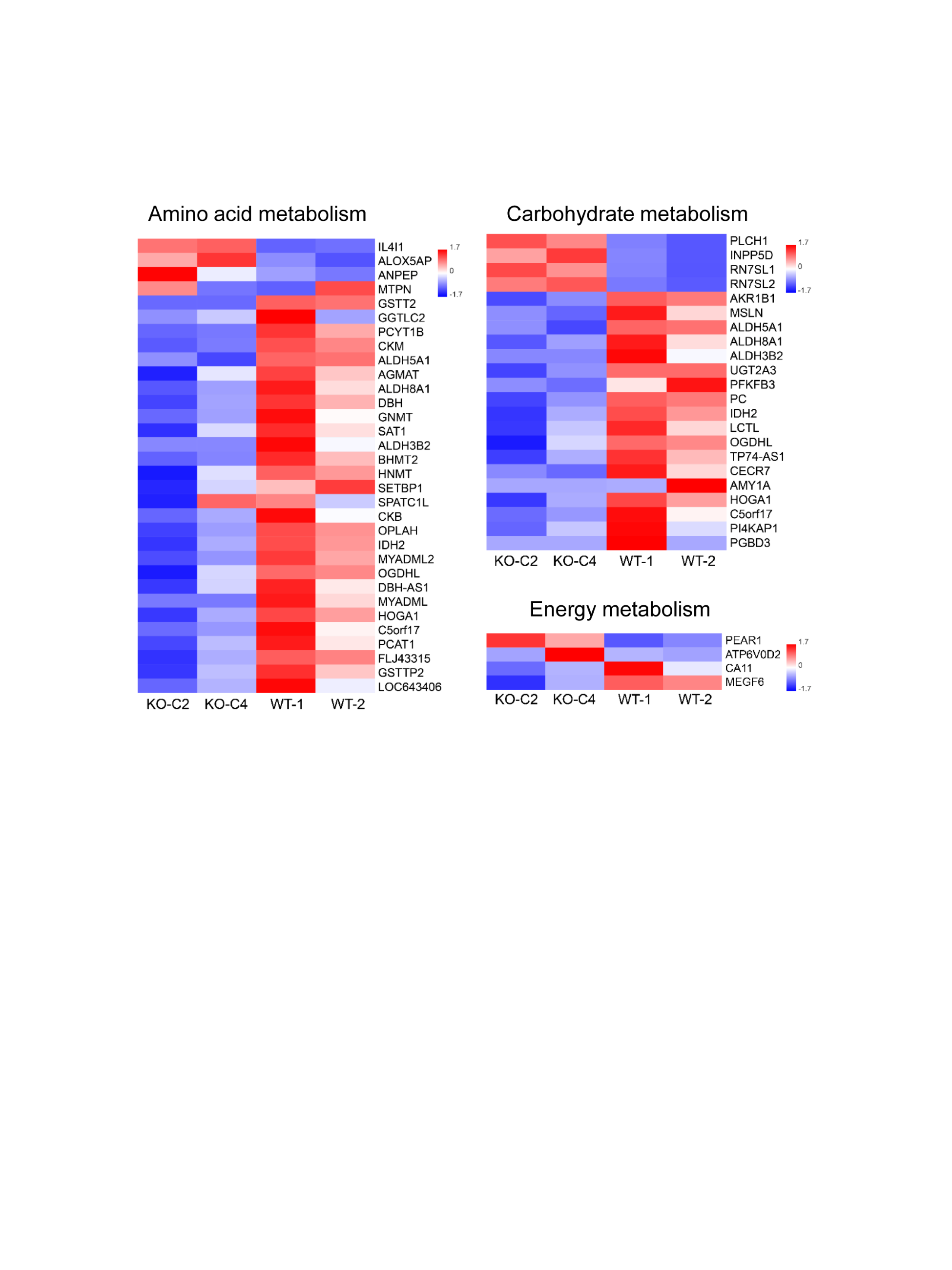

Supplement: S9 Fig — Heat map depicted the enrichment analyses of differentially expressed genes related to amino acid, carbohydrate, and energy metabolism pathways for RNA-seq data from WT and PINLYP KO iSLK-RGB cells. (TIF) [file ppat.1013146.s011.tif]
